# In-Field Detection of Plant Pathogens Using Three-Dimensional-Printed Microneedles and a Portable Platform

**DOI:** 10.1021/acssensors.5c02361

**Published:** 2025-11-27

**Authors:** Emre Ece, Nedim Hacıosmanoğlu, Murat Alp Güngen, Metin Burak Tatlıses, İsmail Eş, Semra Hasançebi, Fatih Inci

**Affiliations:** † UNAM-National Nanotechnology Research Center, 52948 Bilkent University, 06800 Ankara, Turkey; ‡ Institute of Materials Science and Nanotechnology, 52948 Bilkent University, 06800 Ankara, Turkey; § Department of Genetics and Bioengineering, Engineering Faculty, Trakya University, Ahmet Karadeniz Yerleskesi, 22030 Edirne, Turkey

**Keywords:** microneedle-based diagnostics, plant pathogen detection, sustainable agriculture, LAMP, LFA

## Abstract

Plant diseases threaten global food security, necessitating efficient, field-deployable diagnostics. Traditional approaches, including morphological assessments and conventional sampling methods such as swabs or multistep DNA extraction, are often unreliable in the early stages of infection when visible symptoms are absent and subsurface pathogens remain inaccessible, underscoring the need for molecular tools. Here, we present a microneedle (MN)-based diagnostic platform integrated with loop-mediated isothermal amplification (LAMP) and lateral flow assay (LFA), supported by a custom-built portable heater (LAMPbox). The MNs enable the direct extraction of plant fluids and associated pathogens, offering a rapid, simple, and efficient sampling approach. In this study, two MN types were evaluated: poly­(vinyl alcohol) (PVA) and stereolithography-printed MNs fabricated from a plant-derived resin. The 3D-printed MNs exhibited superior mechanical robustness, with a displacement ratio of only 3.3% compared to 24.5% for PVA MNs, and provided an ∼12% higher DNA yield than conventional swab-based methods. Integrated with the LAMPbox, this platform enabled reliable detection of *Puccinia triticina* with clear differentiation between healthy and infected leaves. This work establishes 3D-printed MNs as mechanically robust and effective tools for pathogen sampling while demonstrating the feasibility of a low-cost, portable, and sustainable system for early plant disease diagnosis.

## Graphical abstract

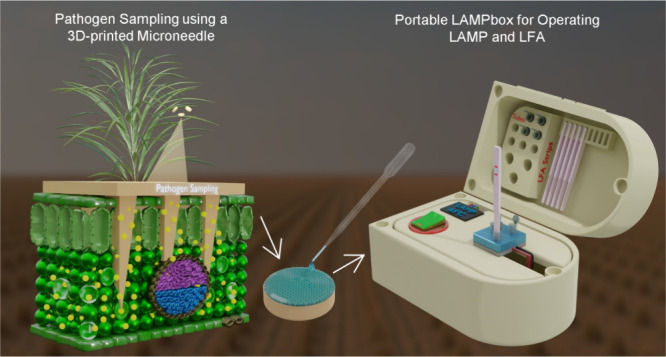

## Introduction

Monitoring and protecting plant health represents a cornerstone of sustainable agriculture, food security, and global economic stability.^1^ Beyond its agricultural and economic significance, ensuring plant health is pivotal for maintaining ecological equilibrium and supporting societal well-being.^2^ In the context of accelerating climate change, rapid population growth, and an increasing demand for food resources, the need to safeguard plant health has never been more urgent. Projections suggest that by 2050, the global population may reach approximately 14.4 billion,^3^ potentially resulting in the loss of up to 1.5 billion metric tons of plant-based resources and an associated economic cost of around $220 billion.^4^ Such scenarios could intensify global resource scarcity and trigger cascading socioeconomic consequences, including widespread job losses, which would further compromise public health and societal resilience. A major threat to plant health arises from phytopathogens, including viruses (*e.g.,* Tobacco mosaic virus (TMV)), bacteria (e.g., *Ralstonia solanacearum*), and fungi (*e.g.,* Puccinia species). These pathogens are disseminated through water, air, soil, and insect vectors, disrupting plant physiology and resulting in significant yield losses. Early and accurate detection of these pathogens is essential, not only to limit their spread but also to enable timely management strategies such as pesticide application and quarantine measures.^5^ Among these, *P. triticina*, a biotrophic fungal pathogen and the causal agent of wheat leaf rust, is particularly destructive. Between 2000 and 2004, this pathogen inflicted an estimated economic loss of $350 million in the United States alone.^6^ Globally, it is responsible for crop yield reductions ranging from 5 to 15%.^7^ Implementing robust early detection systems for *P. triticina* is therefore critical to preserving the wheat yield and ensuring food security for a rapidly growing global population.

Accurate identification of plant pathogens is essential for mitigating disease outbreaks and enabling timely interventions. The diagnostic process typically begins with the collection of symptomatic tissue, such as sections of the stem, root or leaf, which are then transported to centralized laboratories for analysis. Conventional diagnostic techniques, including the cetyltrimethylammonium bromide (CTAB) extraction method and polymerase chain reaction (PCR), are routinely implemented for pathogen detection.^8^ Yet, the transportation of samples to remote laboratories imposes significant costs and delays. During this time, diseases may progress unabated, heightening the risk of pathogen transmission to adjacent crops. Moreover, standard diagnostic protocols are labor-intensive and require complex instrumentation, such as tissue homogenizers, alongside high reagent consumption, which further limits their suitability for rapid, on-site diagnostics. In response to these limitations, the development of low-cost, rapid, and reproducible sampling strategies has gained momentum.^9^ Among the most promising innovations are microneedles (MNs), originally introduced for transdermal drug delivery and biosensing applications.^10–12^ While their applications to plant tissues require a distinct approach due to the fundamental structural and fluidic differences between human skin and plant leaves or other plant organs/sections, the ability of MNs to access subsurface layers—a key advantage in biomedical use—can also be effectively utilized in plants to sample internal tissues. Unlike conventional punches or biopsies, which remove comparatively larger tissue sections and require additional processing, MNs provide a simpler and more direct approach to field sampling, facilitating rapid target recovery without specialized equipment. Leveraging this asset, MNs offer a compact platform that not only enables direct in-field pathogen detection by reducing contamination and enhancing portability but has also been applied in plant health monitoring for the detection of biochemical markers.^13,14^ In particular, the design flexibility of stereolithography (SLA) 3D printing enables fine-tuned customization of MN geometry, density, and patch dimensions to match diverse leaf geometries, offering unique adaptability for plant-focused applications compared to conventional biomedical MN designs.^15–17^ Furthermore, by eliminating the need for specialized PCR systems, MN-based diagnostic platforms have the potential to broaden diagnostic accessibility, enhancing their feasibility for widespread agricultural use.^18^ To be practical for in-field deployment, however, these platforms must generate outputs that can be easily interpreted by non-specialists, end-users, including farmers with minimal technical training. A particularly promising approach involves the integration of LAMP, a technique capable of amplifying nucleic acids under isothermal conditions using simple, portable heating devices.^19^ Coupling LAMP with LFA strips enables the visual detection of amplified DNA with the naked eye, thereby providing a robust, user-friendly diagnostic solution suitable for field conditions.^20,21^

A notable advantage of integrating MNs, portable LAMP systems, and the LFA strategy lies in the ability of MNs to penetrate plant tissues, reaching both surface and subsurface layers. This capability is especially critical for the early-stage detection of pathogens such as *P. triticina*, which initiates infection with urediniospores germinating on the leaf surface but subsequently develops intercellular hyphae and haustoria within the mesophyll tissue. Consequently, *P. triticina* colonizes internal plant tissues rather than remaining confined to the surface, and access to these regions is essential for reliable detection. By providing access to deeper tissue layers, MNs enable efficient extraction of pathogen samples, thereby enhancing diagnostic sensitivity and accuracy.^22^ Furthermore, MN-based sampling serves as a more diagnostically robust approach than conventional morphology-based assessments, which often depend on the visible presence of spores or lesions. In the early stages of infection, when external symptoms have not yet become phenotypically evident, MNs allow for the detection of pathogens before a significant disease progression occurs. This early diagnostic window is critical for initiating timely management interventions and limiting pathogen spread within crop populations.

Several innovative strategies have been investigated to advance agricultural diagnostics, including the integration of smartphone-based portable systems. These platforms utilize the ubiquity and computational capability of smartphones, providing an effective and accessible solution for in-field diagnostics.^23^ Despite this, variability in smartphone camera resolution and device-specific compatibility issues might hinder the standardization and broad scalability. Economically, this poses a burden for low-income farmers who may need to acquire compatible smartphones, further restricting accessibility and widespread adoption. In parallel, dissolvable PVA MNs have been widely adopted in plant studies; nevertheless, their single-use nature poses challenges in terms of sustainability and mechanical reliability.^24,25^ The growing demand for environmentally responsible and economically viable technologies underscores the need for hydrophilic, solid, and mechanically durable MNs. Additionally, conventional DNA extraction protocols, when coupled with LAMP-LFA systems, often require time-intensive and invasive procedures that detract from their practicality in field conditions.^26^ Importantly, few studies have systematically assessed the mechanical robustness of MNs within plant tissues or benchmarked their performance against conventional swab-based sampling for subsurface pathogens, such as *P. triticina*, which colonize internal tissue layers rather than remaining on the surface. Taken together, these limitations collectively emphasize the need for robust, reusable, and mechanically validated MNs, directly compared with conventional methods, to enable reliable and scalable in-field plant pathogen diagnostics.

To address these challenges, we herein present a streamlined, cost-effective, and user-friendly alternative. Our approach combines mechanically durable MNs with an efficient nucleic acid detection workflow, reducing the sample preparation time and procedural complexity. Furthermore, we developed a compact, 3D-printed heating system utilizing readily available materials and simple electronics. This compact heater design aims to expand access to isothermal amplification technologies, particularly in resource-constrained settings. Recent advances in 3D printing have also facilitated the fabrication of intricate biosensing components, including MNs, thereby enhancing the feasibility of rapid prototyping and field deployment in both agricultural and biomedical contexts.^27,28^ By providing a platform that is both affordable (approximately $3 per test in consumable materials and $25 per device) and straightforward to operate and interpret, even for non-specialist users, our strategy offers a scalable solution for agricultural diagnostics. Here, the quoted per-test cost reflects only consumables (resin per MN, LAMP reagents, and LFA strips) and does not include capital or labor costs, which vary greatly across the context. This innovation addresses the main shortcomings of previous approaches and constitutes a significant step toward more sustainable, inclusive, and efficient plant disease monitoring.

In this study, MNs with varying dimensions and materials were fabricated using electrical discharge machining (EDM) and 3D printing technologies. A comparative evaluation was performed to quantify their mechanical strength, hydrophilicity, and reusability (in terms of mechanical performance), focusing on PVA MNs fabricated with EDM-produced titanium molds and resin-based MNs produced via 3D printing. Unlike the dissolvable PVA MNs, the optimized 3D-printed MNs demonstrated improved environmental sustainability, mechanical robustness, and operational efficiency for plant pathogen sampling. PVA and 3D-printed MNs with different sizes were evaluated for their DNA extraction efficiency from *Oryza sativa*, aiming to assess their diagnostic potential in plant pathogen detection. For pathogen detection, MNs were used to capture *P. triticina*, a subsurface fungal pathogen. Following sample collection, DNA amplification was conducted using a portable LAMP system operating at a constant temperature of 65 °C. The amplified products were subsequently visualized using lateral flow assay (LFA) strips, enabling rapid and user-friendly detection without the need for specialized laboratory equipment (Figure 1). In-field validation was performed using integrated LAMP-LFA systems in combination with the optimized MNs. This study is the first systematic comparison between MN-based sampling and traditional swab-based sampling for plant pathogen diagnostics. While swab-based techniques may suffice for surface-residing pathogens, the study highlights that MNs offer distinct advantages for detecting pathogens residing within plant tissues, such as Puccinia spores, where swabbing fails to retrieve sufficient biological material. The results demonstrate that aligning sampling techniques with pathogen localization is crucial for accurate disease detection. Overall, this study presents a cost-effective, portable, rapid, and user-friendly diagnostic platform suitable for in-field plant disease monitoring. By benchmarking the MN-based system against conventional sampling methods, this work lays the groundwork for the broader implementation of MN and portable molecular diagnostics in sustainable agriculture.

**Figure 1:**
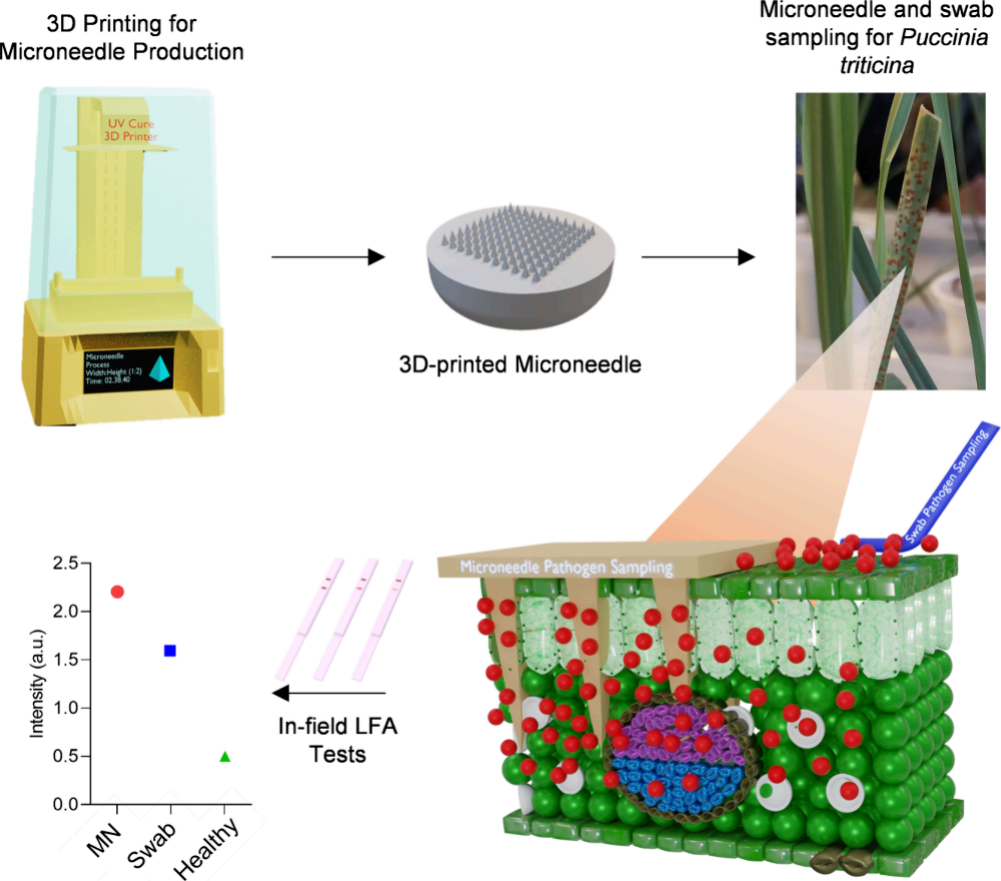
Workflow of MN implementation for the portable plant pathogen detection system.

## Experimental Section

### Fabrication of Titanium and PVA MNs

To fabricate PVA MN molds, AutoCAD software was employed to design the small (450 μm in height) and large (700 μm in height) titanium MNs. In these designs, the base width and height were defined as 300 and 450 μm for the small titanium (225 needles per patch), while these values were 350 and 700 μm for the large titanium MN (376 needles per patch) (Figure S1). The fabrication process was performed using an EDM device capable of micron-level precision. Electrically heated wires enabled the formation of MN molds through controlled melting and erosion. In the subsequent step, polydimethylsiloxane (PDMS) negative molds were produced using these titanium positive molds. To achieve this, first, the titanium molds were taped with Kapton tape, and PDMS and curing agent solutions, prepared at a ratio of 10:1 (v:v), were poured onto the molds. The curing process was carried out at 120 °C for 30 min. Afterward, these negative PDMS molds were again taped with Kapton tape to serve as templates for producing the PVA MNs. Solutions with concentrations of 2.5, 5, 7.5, 10, 12.5, and 15% (150 mg in 1 mL) were cast into each mold type, and PVA MN fabrication was completed following a 36 h drying period at room temperature (Figure S2).

### Fabrication of 3D-Printed MNs

Designs of small and large 3D-printed MNs were completed using Shapr3D software. Small MNs (121 needles per patch) were designed with a width of 500 μm and a height of 1000 μm, while the width and height for the large MNs (121 needles per patch) were set to 750 and 1500 μm, respectively. To evaluate the effect of printing angle and curing time on hydrophilicity and mechanical strength, respectively, 0, 45, and 75° printing angles and 10, 20, and 30 min curing times were applied to each MN design (Figure S3). The printing process of these MNs was completed using an SLA-based printer (Halot One CL-60, Creality, China) with a 2.2 s exposure time, 40 s base layer exposure time, and 25 μm *Z*-axis layer resolution. After printing, the MNs were rinsed using isopropyl alcohol (IPA) for 30 min and post-cured under UV light at 405 nm.

### Chemical Characterization of PVA MNs

The attenuated total reflectance–Fourier transform infrared spectroscopy (ATR-FTIR) (Thermo Fisher, USA) technique was implemented for the chemical analysis of PVA powder and PVA MNs to evaluate the protection of hydroxyl groups (OH), making the PVA hydrophilic, in the PVA structure during the MN production process. First, both powder and MN samples were dried at 50 °C for 2 h. The dried samples were analyzed after cleaning the ATR-FTIR diamond crystal surface with IPA.

### Contact Angle Measurements of MNs

In the first part, the effect of solution concentration on PVA hydrophilicity was evaluated using contact angle measurement (Dataphysics OCA 30, Germany). Following that, the effect of the printing angle on the hydrophilic behavior of 3D-printed MNs was examined using the same procedure. In both processes, after dropping 3 μL of water on the surface of MN, the image was taken after waiting 15 s (sampling time) using SCA200 software to calculate the contact angle. Five replicates were carried out for each MN for these measurements. Then, the hydrophilicity of MNs was analyzed by using a violin bar chart in GraphPad software.

### Scanning Electron Microscopy (SEM) and 3D Laser Scanning Microscope Analyses of MNs

First, an SEM (FEI Quanta 200F, USA) instrument was operated for the morphological and dimensional analyses of MNs. To prepare the samples, PVA and 3D-printed MNs were coated with 10 nm palladium:gold (Pd:Au) using a precision etching coating system (PECS) (Gatan 682, USA), making their surface conductive for SEM analysis. Before the analyses of PVA MNs, their titanium molds were examined first to establish a standard for comparison. All MNs and molds were measured with a 30° tilt angle under 15 kV and 3.0 spot power to enhance the visibility of the MN morphology details. The same experimental procedure was also applied to the PVA and 3D-printed MNs after dynamic mechanical analysis (DMA) measurements to assess the effect of 18 N compression on the MNs.

A 3D laser scanning microscope (Keyence VK X100, Keyence, Japan) was operated for geometrical analyses of titanium, PVA, and 3D-printed MNs. In the first part, a comparison between titanium and PVA MNs was carried out to evaluate the fabrication performance of EDM and height/width loss in PVA MNs. The size differences between the Shapr3D design and the 3D-printed MNs were also evaluated using the same procedure. In the measurement part, a 10× objective lens and 200 nm *Z*-axis step size parameters were selected for all MNs. After scanning, the results of the 3D model were analyzed using VK-Analyzer software.

### Mechanical Analysis of MNs

A compression test with an 18 N static force was performed for each MN using DMA (TA Instruments – Q800, USA). The mechanical strength tests on PVA MNs were conducted, and the effects of the MN concentration and geometrical properties on mechanical strength were systematically investigated. On the other hand, the effects of printing angle, curing time, and size differences on 3D-printed MNs were scrutinized. For these analyses, an 18 N static force was applied to the MNs, increasing by 0.5 N/min, and the process was carried out at room temperature. The displacement ratio per N/needle results were evaluated. As a result, the optimal concentration that provided the best mechanical strength for PVA MNs was selected for two different sizes, while the optimal curing times and printing angles for each size of 3D-printed MN were determined. One of the optimum-performing MNs was also subjected to ten consecutive compression tests at 18 N with a loading rate of 18 N/min to observe the effect of repeated measurements on tip integrity and overall geometry.

### PCR Analysis for *Oryza sativa* Using Conventional, Swab, and MN Sampling

The online tool Primer Blast was utilized to design the PCR primers for the target DNA sequence of *Oryza sativa* plant using the design standards and the literature (Table S1).^29^ To obtain a standard DNA solution of that target sequence for PCR primer confirmation and carry out the performance comparison between swab and MN sampling, the conventional CTAB method was employed (Figure S4). Before MN implementation for the DNA extraction, swab sampling was performed from the 1 cm × 1 cm surface area of the leaf using a pipette. After this sampling, the surface of that pipette was rinsed, and the solution was stored for further PCR experiments. In the MN sampling process, the most optimal MNs from each type (PVA and 3D-printed) and each size (small and large for both types) were manually pressed into the leaf tissue by hand and held in place for approximately 15–20 s. During this time, the MNs remained physically anchored, allowing plant fluid to adhere to their surfaces through natural adhesion. The penetration step also caused limited cell disruption, thereby releasing intracellular contents, including plant DNA, into the surrounding fluid, ensuring that the captured sample represented the leaf tissue. After that, the rinsing process of the surface of MNs was completed by using 100 μL of ddH2O to collect the extracted DNA samples (Figure S5). After collecting the DNA samples using all these sampling methods, the PCR setup was prepared and operated. In PCR assays, LUNA Universal qPCR Master Mix (NEB, USA) was used following the standard protocol, and the PCR analysis was performed using CFX96 (BioRad, USA). Following that, Cq values and log DNA concentrations (DNA/mL) were calculated from a serial dilution series and samplings, and a standard PCR curve was generated. Lastly, statistical analysis was conducted using Student’s *t* tests in GraphPad software.

### LAMP and LFA analyses for *Puccinia triticina*

The LAMP primers are derived from the diagnostic primers, Puc.ITS that were designed using standard parameters with the Primer Explorer 5 program (https://primerexplorer.jp/lampv5e/index.html) (Table S2). In LAMP primers, TTTT was included as a linker, following a previous report.^30^ The susceptible (Morocco) and resistant (Tc*Lr24) wheat seedlings were inoculated with fresh *P. triticina* urediniospores (Figure S6).^31^ The DNAs were extracted from the infected wheat leaves and urediniospores by conventional Norgen’s plant/fungi DNA isolation kit, swab, and MN sampling techniques, all performed on comparable 1 cm × 1 cm leaf areas to ensure consistency and allow direct performance comparison. Importantly, these three approaches were evaluated independently rather than sequentially on the same leaf samples to avoid any potential cross-interference between methods. After obtaining DNA from the plant pathogen using a conventional method as described in the literature,^32^ the swab sampling was completed three times per day for susceptible leaf and resistant leaf samples, as described in the *Oryza sativa* experiments. Similarly, MN sampling was conducted three times using the small 3D-printed MNs. For this study, each MN was used only once per sampling event to completely avoid possible cross-contamination for disease diagnostic purposes. During insertion, the MNs penetrated the leaf epidermis and mesophyll, where the plant fluid adhered to their surfaces through natural adhesion. This fluid contained both the host material and the pathogen. In detail, measurements were taken from three leaves of each plant species each day. After the measurements were completed with swabs and MNs for the 1st, 2nd, 3rd, 4th, 5th, and 15th days, samples were taken from non-inoculated leaves for positive control purposes. In each sampling, as performed in the PCR section, the pathogen samples obtained were stored by washing the sampling surfaces with 100 μL of ddH2O. Moreover, to verify that no measurable inhibition was introduced by the 3D-printed material, control LAMP reactions were performed with DNA samples that had been pre-exposed to cured resin MNs. WarmStart Colorimetric LAMP 2× Master Mix (NEB, USA) was used throughout the work for LAMP reactions following the manufacturer’s recommendations. LAMP Fluorescent Dye (NEB, USA) was also used to track LAMP reactions in real-time with a BioRad CFX96 real-time PCR device.

After LAMP reactions, LFA strips (Milenia Hybridetect, Milenia Biotec GmbH, Germany) were placed in 50 μL of the reaction mixtures in each well. LFA strips were kept in these wells for 10 min at room temperature to observe changes in the test and control lines. Afterward, the strips were both visually inspected, and photographs of them were taken using a smartphone (Samsung Galaxy A23). These photographs were then analyzed to detect line thickness with pixel intensity using ImageJ software.

### DNA Amplification and LFA Detection

DNA amplification of the pathogen and LFA detection mechanism of the system begin with the addition of pathogen-infectied leaves to a reaction mixture comprising reaction buffer, Bst DNA polymerase, and dNTPs. After incubation at 65 °C, the inner primers (FIP and BIP) hybridize to the target region and initiate the synthesis of a new strand while simultaneously forming loop structures. Subsequently, the outer primers (F3 and B3) anneal to the newly synthesized strand and displace the loops, thereby creating additional binding sites for the LF and LB primers, which further accelerate the overall amplification process.

For detection, hybrid primers labeled with biotin and FITC were employed, resulting in the generation of amplified hybrid DNA molecules carrying both labels. Following amplification, the products were applied to an LFA strip functionalized with streptavidin (test line) and anti-goat IgG antibody (control line). During migration along the strip, the amplified DNA molecules interact with colloidal gold nanoparticles (NPs) conjugated to anti-FITC antibodies. In the presence of amplification, streptavidin captured the biotin-labeled hybrids, while the FITC moiety was recognized by the gold-conjugated anti-FITC antibody, producing visible bands at both the test and the control lines. In the absence of amplification, only the control line appears, as the colloidal gold-anti-FITC conjugates are retained at the anti-goat IgG control region.

### Agarose Gel Electrophoresis

1% Agarose gel containing 1× SYBR Safe DNA gel stain (Thermo Fisher, USA) was prepared in 1× Tris-acetate-EDTA (TAE) buffer to run LAMP samples with 6× gel loading dye (Thermo Fisher, USA) and was visualized using a standard transilluminator.

### Production and Implementation of Portable Heater

First, we set up the system using Arduino Nano and the sensor shield. A metal-oxide-semiconductor field-effect transistor (MOSFET) was used to regulate the 5 V 8 W positive thermal coefficient (PTC) heater. One of the Arduino Nano’s pulse-width modulation (PWM) pins (D3) activated the MOSFET. The HT-NTC 100 K 3 mm thermistor was utilized to measure the temperature. This thermistor was connected via a voltage divider circuit to the Arduino’s analog input pin (A0). One end of the thermistor was connected to +5 V, and the other end was connected to a 100 kΩ resistor, which was then connected to GND. The analog input pin was linked to the junction of the resistor and thermistor. The LED display was connected to an Arduino following the I2C communication protocol and using the SDA and SCL pins. Our Arduino code takes the information from the thermistor, computes the temperature, and shows the result on the LED screen. At the same time, the code uses a proportional–integral–derivative (PID) controller to control the heater, heating the heat block up to then maintaining its temperature at 65 °C.

## Results and Discussion

### Characterization of Titanium and PVA MNs

Titanium MN molds with two sizes were fabricated via EDM. Residual machining oils were removed by sonication in 50% acetone for 1 h, followed by PDMS casting (10:1 w/w base-to-curing agent) to form negative molds. PVA MNs were then cast into the PDMS molds at six different concentrations, generating 12 MN groups in total. Chemical characterization using ATR-FTIR demonstrated the preservation of hydroxyl groups essential for hydrophilicity, as evidenced by the 3293 cm^–1^ O–H peak in both processed and unprocessed PVA spectra (Figure S7A). Increasing the PVA concentration resulted in enhanced hydrophilicity, with contact angles decreasing from 61.5 ± 2.3 (2.5%) to 37.4 ± 2.1° (15%) (Figure S7B). Morphological analysis by SEM and Keyence microscopy (Figure 2A,B) revealed a high replication fidelity between titanium molds and PVA MNs. The small titanium MNs closely matched the AutoCAD design (450 μm height, 350 μm width) with measured values of 449.6 and 352 μm, respectively, and a tip diameter of 18 μm. The corresponding PVA MNs showed minor shrinkage after drying (432.3 μm height, 307.1 μm base width, 18 μm tip). Similarly, large titanium MNs (700 μm height, 350 μm base width, 9.2 μm tip) produced PVA counterparts measuring 650.7 μm in height, 327.7 μm in base, and 13.2 μm tip. The high precision achieved by EDM confirmed its suitability for mold fabrication; however, due to its high operational cost and limited throughput, alternative approaches such as 3D printing were adopted to improve production efficiency. Although 3D printing cannot fully replicate EDM’s resolution, the 3D printing strategy provides a practical route for rapid and scalable mold fabrication. These initial results validated the structural fidelity and hydrophilic tunability of PVA MNs, which are crucial for proper plant fluid uptake during DNA extraction. Nevertheless, despite their excellent replication quality, PVA’s polymeric nature causes mechanical and dimensional instability upon dehydration, making it suboptimal for repeated sampling. This limitation prompted a transition toward resin-based 3D-printed MNs, which combine precise geometry with superior mechanical resilience.

**Figure 2:**
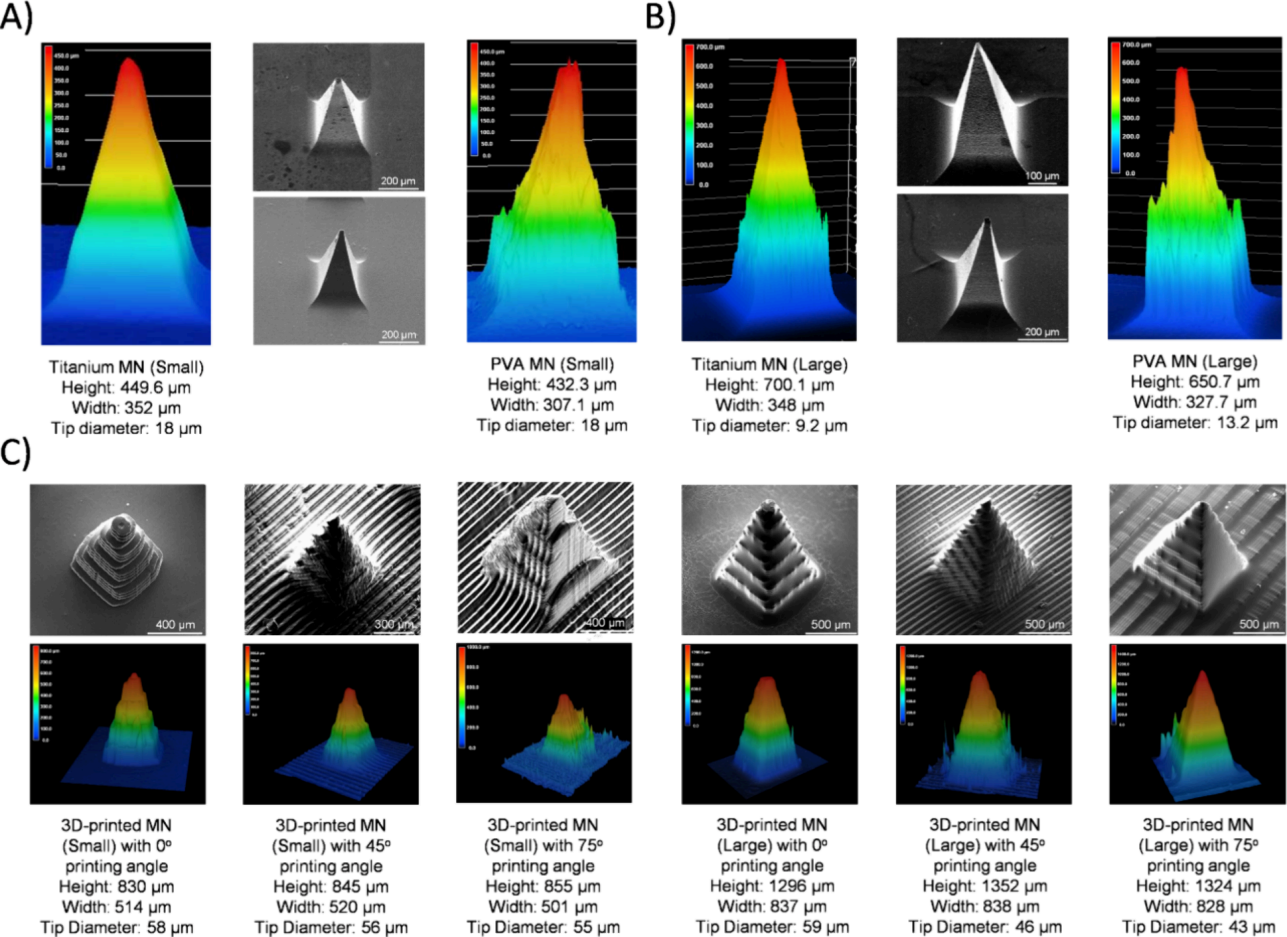
PVA and 3D-printed MNs were geometrically and morphologically characterized. (A) Keyence and SEM analyses of small titanium and PVA MNs were performed to assess their structural fidelity and tip morphology. (B) Keyence and SEM analyses of large titanium and PVA MNs were conducted to compare height, base width, and tip sharpness. (C) 3D-printed MNs fabricated at 0, 45, and 75° printing angles (small and large sizes) were analyzed using SEM and 3D profilometry to evaluate the effects of printing angle and geometry on structural accuracy and tip definition.

### Characterization of 3D-Printed MNs

Resin-based MNs were fabricated via two different sizes with same aspect ratios (1000:500 and 1500:750 μm (height:base)) and three printing angles (0, 45, and 75°) (Figure S7C). Each sample underwent postcuring for 10, 20, and 30 min to evaluate the influence of printing orientation and curing duration on mechanical properties and surface wettability. By maintaining the same aspect ratio across the two sizes, the influence of printing angle and curing time on both mechanical strength and surface hydrophilicity was systematically assessed. Hydrophilicity, a critical factor for plant fluid sampling, was quantified through contact angle measurements: 58.4° ± 4.4° (0°), 74.5° ± 6.3° (45°), and 77.6° ± 6.4° (75°) (Figure S7D). An increase in contact angle corresponded to reduced wettability, associated with higher surface roughness, thereby demonstrating the influence of printing angle on fluid interaction.

Geometric fidelity was characterized by SEM and Keyence microscopy. For small MNs, printed heights were 830 (0°), 845 (45°), and 855 μm (75°); base widths were 514, 520, and 501 μm; and tip diameters were 58, 56, and 55 μm, respectively. The observed ∼16% height reduction is consistent with limitations of *Z*-axis resolution and resin shrinkage, whereas the ∼4% base overgrowth was attributed to excess lateral curing. For large MNs, printed heights were 1296 (0°), 1352 (45°), and 1324 μm (75°); base widths were 837, 838, and 828 μm; and tip diameters were 59, 46, and 43 μm. Higher printing angle produced sharper tips by aligning final printed layers with the *Z*-axis, thereby improving vertical resolution (Figure 2C).^33^

Although EDM molds provided higher dimensional precision, SLA printing enabled faster, more economical fabrication (∼150 MNs day^–1^ at ≈$0.10 each) and facilitated rapid design iteration. Moreover, the resin MNs exhibited non-solubility and mechanical durability, providing sustainable and reusable alternatives for field-based agricultural diagnostics.

### DMA Analysis of MNs

Mechanical strength plays a key role in the MN performance, especially for both penetration efficiency and reusability. High mechanical integrity not only preserves MN geometry during insertion but also minimizes the risk of breakage and residual material remaining in the target tissue. To evaluate mechanical performance, compression tests were first performed on both small (225 needles) and large (376 needles) PVA MNs fabricated at varying polymer concentrations. Displacement values (μm), displacement ratios (%), and normalized force values (N/needle) were determined following the application of the 18 N compression force. In both MN sizes, an increased polymer concentration resulted in enhanced mechanical strength. For instance, a small PVA MN at 2.5% concentration (height: 430 μm) exhibited a displacement of 364 μm, corresponding to an 84.6% displacement ratio, whereas at 15% concentration, the ratio decreased significantly to 34.2% (Figure 3A). Across all concentrations, the maximum per-needle force remained stable at ∼0.08 N/needle. Similar trends were identified in the large PVA MNs (Figure 3C), where displacement declined from 43.4% to 24.5% as the concentration increased from 2.5% to 15%. In the case of large PVA MNs, the maximum per-needle force remained constant at ∼0.048 N/needle across all concentrations. This enhancement in strength corresponds to the increased polymer chain density in more concentrated solutions, which more effectively resists compressive deformation. Despite the improved mechanical performance, SEM analysis revealed notable tip deformation under load (Figure 3B–D). Although small PVA MNs have shorter heights, they consistently exhibited higher displacement ratios than large PVA MNs due to their greater force per needle (∼0.08 vs ∼0.048), which caused more pronounced deformation. Because the inter-needle spacing in both small and large arrays was nearly identical, the taller geometry of the large PVA MNs produced a denser and stiffer array that better resisted compressive forces.

**Figure 3:**
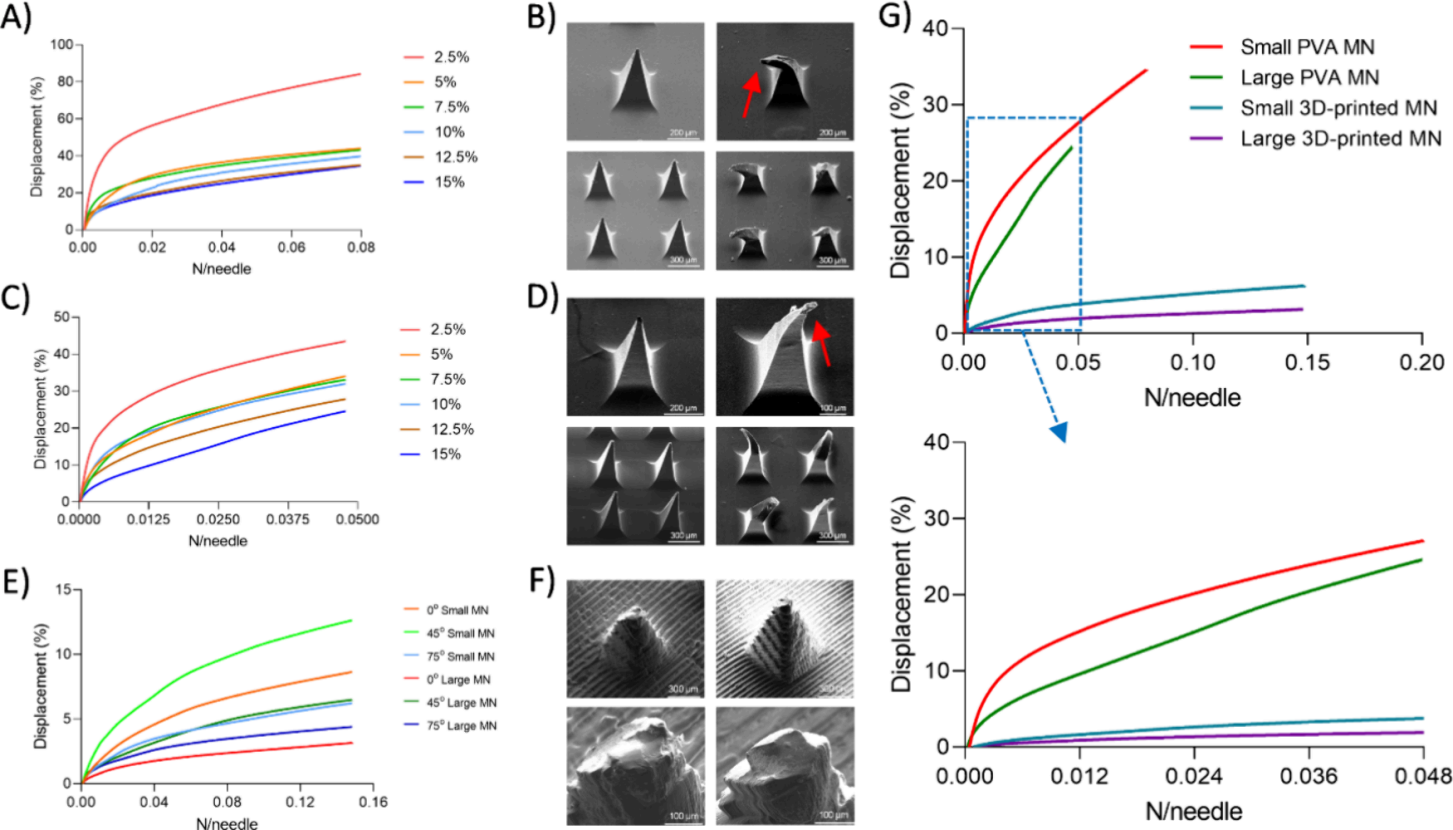
(A) Small PVA MNs and (C) large PVA MNs with varying concentrations were mechanically analyzed to assess their displacement ratio under their N/needle values. (B) Small PVA MNs and (D) large PVA MNs were examined morphologically using SEM to observe structural changes following DMA testing (red arrows indicate bent or deformed tips). (E) 3D-printed MNs of different sizes (1000 and 1500 μm in height) and printing angles (0, 45, and 75°) were analyzed using DMA to evaluate the effects of size and angle on mechanical strength. (F) The impact of an 18 N static force on the tips of 3D-printed MNs was assessed by using SEM to investigate structural deformation. (G) Comparison of the best-performing MNs from each type, highlighting the differences between PVA and 3D-printed MNs.

Subsequently, 3D-printed MNs were evaluated under the same mechanical conditions for internal comparison and benchmarking against PVA MNs (Figure 3E). A total of 18 3D-printed MNs, which are in two sizes, three printing angles (0, 45, and 75°), and subjected to three postcuring durations (10, 20, and 30 min), were analyzed. Each array consisted of 121 needles, and both small and large designs reached a similar maximum force of ∼0.15 N per needle under the 18 N compressive force. Among curing times, 30 min yielded the highest mechanical strength across all samples, which can be attributed to the more complete photopolymer cross-linking achieved during extended UV exposure. Longer curing enhances the degree of polymer network formation and reduces residual unreacted monomers, thereby increasing stiffness and reducing plastic deformation under load.^34^ For example, in small MNs printed at 0°, displacement ratios decreased with longer curing: 15.0% (10 min), 12.1% (20 min), and 9.6% (30 min). Similarly, for large MNs printed at 75°, displacement dropped from 6.1% to 4.7% across the same curing intervals (Figures S8–S10). The impact of printing angle was also significant. In small MNs, 75° printing yielded the highest mechanical strength, with a displacement ratio of 6.9%, whereas 45° printing showed the lowest performance (∼13.8% displacement). In large MNs, the 0° printing angle was optimal, yielding a displacement ratio of ∼3.3% (Figure 3E). The reduced strength observed at 45° across both sizes was likely due to layer orientations being misaligned with the compressive force direction, limiting efficient load transfer and back-force generation. Although both small and large 3D-printed MNs have the same needle count and N/needle values, the small MNs consistently exhibited greater displacement. This can be attributed to the geometric effect: (i) in the large MNs, the needles are taller while maintaining nearly same interspacing, leading to a stiffer and more compact architecture. (ii) In contrast, the small MNs appear more spread out, which allows greater lateral compliance under compressive load. Additionally, the effect of printing angle is size-dependent. In small MNs, the 75° orientation might enhance tip sharpness and reduce interlayer shear, thereby maximizing strength. However, in large MNs, the increased height amplifies interlayer mismatch at 75°, which might lead to potential shear and buckling. Therefore, 0° printing, where the stacking direction could be perfectly aligned with the needle axis, provides a more column-like structure and the highest stability for taller MNs.

Compared with PVA MNs, the 3D-printed MNs exhibited superior mechanical properties across all conditions. This advantage is attributed to both the inherent stiffness of the photopolymer resin and the improved cross-linking achieved through optimized curing. SEM analysis supported these findings; while PVA MNs showed significant tip bending, 3D-printed MNs exhibited minimal deformation (Figure 3F), suggesting that observed displacements were largely due to elastic deformation rather than structural failure. When the best-performing MNs from each group were directly compared, clear differences in mechanical behavior were observed. Small PVA MNs (225 needles, 0.08 N/needle) exhibited the lowest displacement ratio (∼34.2%), followed by large PVA MNs (376 needles, ∼0.048 N/needle) with 24.5%. In contrast, both small and large 3D-printed MNs (121 needles, 0.15 N/needle) demonstrated much lower displacements (6.9 and 3.3%, respectively), indicating that despite being subjected to higher forces per needle, they resisted deformation more effectively. Importantly, repeated compression testing revealed that 3D-printed MNs maintained their structural integrity over ten consecutive cycles, with displacement stabilizing after the first few tests and no cumulative tip damage observed. This suggests a high level of fatigue resistance, supporting their potential for limited reusability in terms of mechanical stability (Figure S11). These results highlight that the superior stiffness of 3D-printed MNs originates from their densely cross-linked acrylate resin network, which forms a stable and rigid polymer matrix with reduced viscoelastic creep compared to that of the semicrystalline PVA structure. The strong covalent cross-linking within the resin prevents localized yielding and enables elastic recovery under compressive load, thereby maintaining geometric integrity during repeated use (Figure 3G). Similar performance advantages have been reported in SLA-printed MNs designed for interstitial fluid sampling of C-reactive protein (CRP) and procalcitonin, where displacement ratios below 3% around 0.08 N/needle.^35^ Such mechanical robustness is essential for repetitive plant tissue penetration, ensuring a consistent insertion depth without tip blunting or permanent deformation. Taken together, the results demonstrate that resin-based MNs possess both mechanical resilience and structural fidelity, rendering them well-suited for field-deployable sampling applications. Owing to their superior mechanical and geometric stability, the optimized 3D-printed MNs were selected, along with PVA counterparts, for comparative DNA extraction studies using *Oryza sativa* leaves.

### Quantitative PCR (qPCR) Analysis of *Oryza sativa* Using Swab and MNs

The DNA extraction applications of the optimal four different MNs on *Oryza sativa* were carried out, as shown in Figure 4A, respectively. After sampling with these MNs, a swab sample was taken from a plant surface that had not been treated with an MN. The MN and swab surfaces were washed with 100 μL of ddH_2_O, and the captured DNAs were collected and stored for PCR analyses. In the first stage of the analysis section, DNA extraction of the *Oryza sativa* plant was completed using a conventional CTAB method. A calibration graph of log­(concentration) versus Cq values was created using the PCR results for the obtained DNA concentrations of 100, 10, 1, and 0.1 ng/μL (Figure 4B). Homogenization with CTAB extraction is a gold standard for targeted nucleic acid recovery. On the other hand, our study focuses on the development and validation of a field-deployable sampling and detection platform; the most relevant comparison was with other portable methods, such as surface swabbing. As expected, no DNA extraction was observed in the swab sampling since the internal plant fluid is not accessible through this technique. DNA extraction was achieved at concentrations of 0.29 ng/μL, 1.43 ng/μL, 1.03 ng/μL, and 0.37 ng/μL with small PVA, large PVA, small 3D-printed, and large 3D-printed MNs, respectively (Figure 4C). However, due to variations in MN sizes and the number of needles per patch, calculating DNA extraction efficiency in terms of nanograms of DNA per mm^3^ and per needle offers a more precise method for identifying the most effective MN.

**Figure 4:**
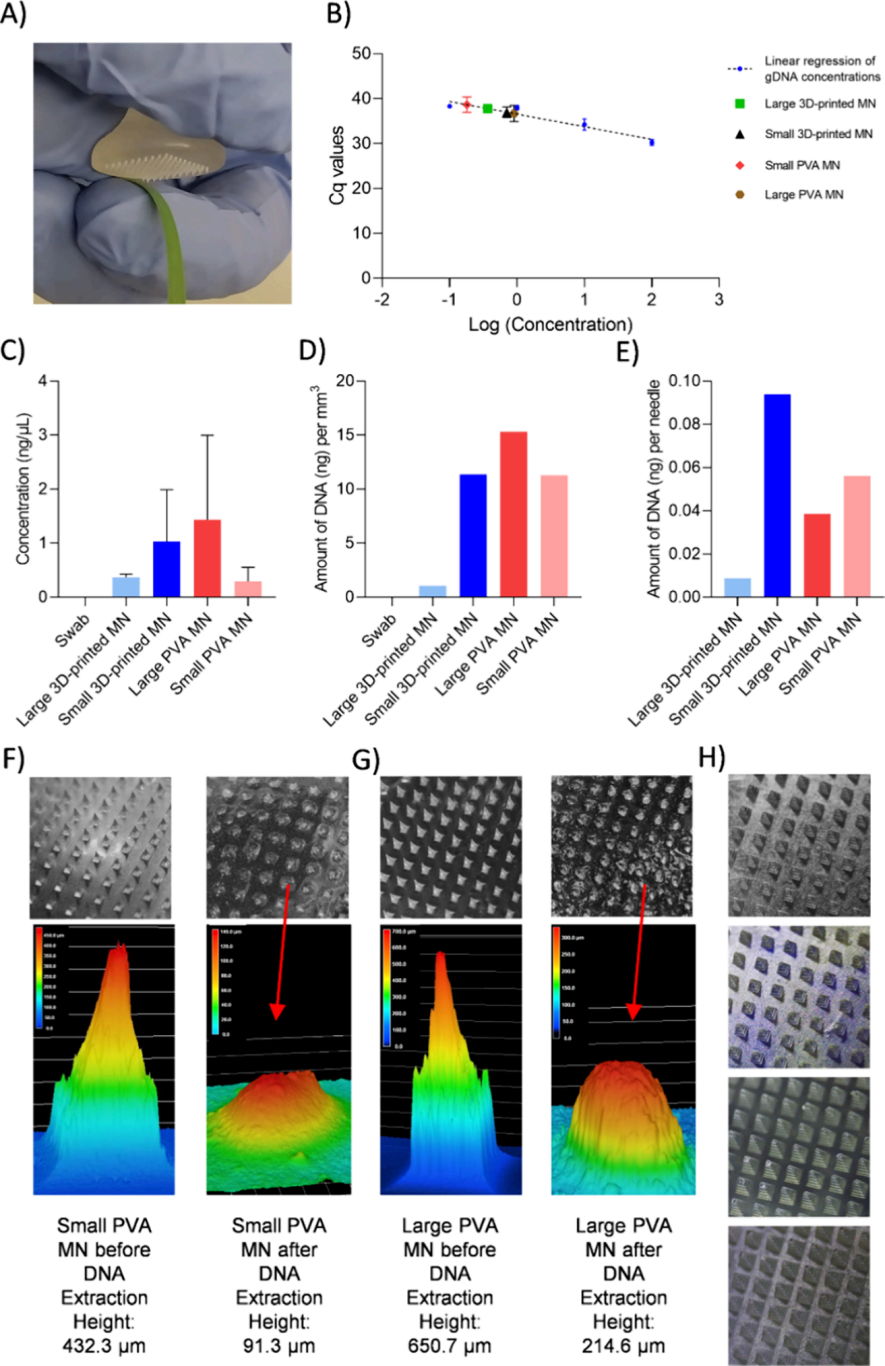
PVA and 3D-printed MNs were used for DNA extraction from *Oryza sativa*. (A) An optical image was presented to demonstrate the MN application on the plant for DNA extraction. (B) PCR results were analyzed for small PVA, large PVA, small 3D-printed, large 3D-printed MNs, as well as swab and the conventional CTAB method. (C) The total concentration of DNA extracted using swabs and MNs was examined, with the large PVA MN yielding the highest DNA concentration. (D) The volume-based amount of extracted DNA was compared between the swab and MN methods. (E) The DNA extraction efficiency per needle was calculated, showing the highest efficiency with the small 3D-printed MN. (F) Small PVA MNs and (G) large PVA MNs were assessed morphologically using microscopy (top) and Keyence imaging (bottom) to illustrate the significant structural changes after DNA extraction (red arrows indicate dissolved needle tips). (H) Optical microscopy images of 3D-printed MNs after extraction. The upper two images indicated small 3D-printed MNs, while the lower two images exhibited large-sized 3D-printed MNs.

The highest extraction efficiency per unit volume was achieved with the large PVA MN (15.31 ng/mm^3^), benefiting from its larger geometry and high needle density (376 needles; volume: 9.98 mm^3^) (Figure 4D). This apparent advantage, however, primarily reflects the substantially higher number of needles on the large PVA patch compared with 3D-printed patches (121 needles). When normalized on a per-needle basis, the highest DNA yield per needle was recorded for the small 3D-printed MN (0.09 ng/needle), which contained 121 needles and a total volume of 9.07 mm^3^ (Figure 4E). The results demonstrate that small 3D-printed MNs exhibited a higher sampling efficiency per penetration point, and increasing their density to levels comparable with large PVA MNs would further enhance total DNA recovery. The superior performance of small 3D-printed MNs relative to their large counterparts may be explained by their greater localized interaction with plant tissue, whereas larger MNs tend to puncture deeper and pass through more readily, reducing fluid retention. A similar effect was observed in PVA MNs, where small patches showed higher per-needle efficiency than large ones, likely due to their broader geometry and reduced tendency to fully perforate the tissue. The relatively lower efficiency of the large 3D-printed MN was attributed to excessive tissue damage. Its increased penetration force likely led to full perforation of the plant cell wall, causing fluid leakage onto surrounding tissues rather than effective absorption into the MN array.

In terms of structural resilience and reuse potential, 3D-printed MNs demonstrated a superior mechanical integrity. Optical images and Keyence measurements revealed that both small and large PVA MNs experienced severe tip deformation and reduction in height after a single use (Figure 4F,G), compromising their penetration capacity in subsequent applications. Furthermore, although PVA MNs possess inherent hydrophilicity, their partial dissolution during rinsing diminished the per-needle DNA extraction efficiency. While such dissolution could theoretically enhance DNA release into the washing buffer, in practice, it poses major drawbacks, including reduced mechanical stability, difficulty in standardizing extraction volumes, and risks of assay inhibition from polymer residues. In contrast, solid 3D-printed MNs maintained their geometry after rinsing, enabling reproducible sampling and reliable downstream analysis. This mechanical retention is particularly important for repetitive or in-field testing, as consistent geometry ensures uniform penetration depth and reproducible extraction volume. Collectively, these results establish the small 3D-printed MN as the most effective and mechanically robust option for plant DNA sampling, outperforming traditional PVA MNs in both functional and mechanical metrics (Figure 4H). The cross-linked resin network underlying these MNs provides a stable, nondeforming substrate that preserves tip integrity, enabling reliable sampling from internal tissues. This optimized MN design was subsequently employed for the selective extraction and detection of the plant pathogens.

### Detection of *P. triticina* Using the Unified Platform (3D-Printed MNs, Portable LAMP, and LFA)

In the initial phase of pathogen detection, sampling was performed on days 1, 2, 3, 4, 5, and 15 dpi (days post-inoculation) of both the susceptible and resistant wheat leaves using the optimized small 3D-printed MNs and conventional swabs. Prior to these time-course samplings, a reference DNA extraction from *P. triticina* urediniospores was carried out using the traditional method to evaluate how variations in pathogen DNA concentration affect LFA output (Figure 5A). Following extraction, *P. triticina* DNA was quantified and diluted to concentrations of 40 ng/μL, 4 ng/μL, and 0.4 ng/μL. These DNA samples were then amplified via LAMP, and the resulting amplicons were visualized using LFA strips. In parallel, control samples were collected from healthy, non-inoculated plants by using both swabs and MNs (Figure S12). The LFA results obtained from both the traditional DNA extraction method and control samplings were used to establish a correlation between DNA concentration and LFA signal intensity. This relationship was quantified using eq 1, providing a calibration framework for interpreting the semiquantitative LFA outcomes in subsequent MN-based field sampling experiments.

$$\mathrm{LFA}\;\mathrm{value}=\frac{\mathrm{test}\;\mathrm{line}\;\mathrm{value}}{\mathrm{control}\;\mathrm{line}\;\mathrm{value}}$$ 1

**Figure 5:**
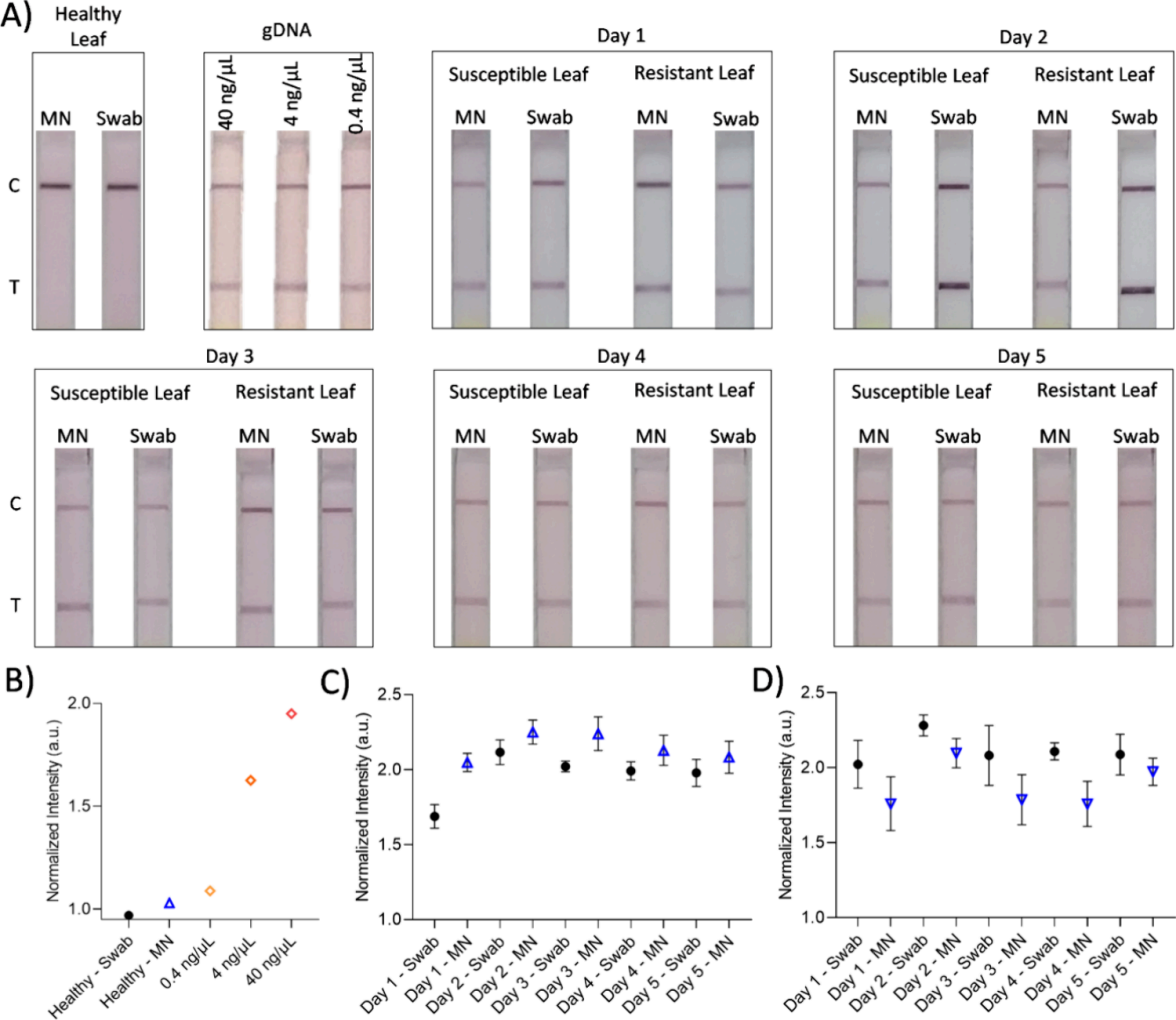
LFA analyses of swab and 3D-printed MNs for *P. triticina* detection were demonstrated. (A) LFA results of gDNA extracted using swabs and MNs over a 5 day period (days 1 to 5) were exhibited, alongside results from healthy leaves and susceptible and resistant wheat leaves. (B) Image analyses of LFA results for healthy leaf samplings using swab and MN and gDNA using conventional method were demonstrated. (C) Infected susceptible leaves and (D) infected resistant leaves were sampled over a 5 day period using swabs and MNs to evaluate the efficiency of MNs in detecting *P. triticina*. Data are presented as mean ± SD (*n* = 3 per group). Statistical analyses were performed using two-way ANOVA followed by Bonferroni’s multiple comparison test (*n* = 3, *p* < 0.05). Significant main effects were observed for both sampling method and duration in susceptible (method: F­(1, 20) = 38.84, *p* < 0.0001; duration: F­(4, 20) = 12.31, *p* < 0.0001) and resistant (method: F­(1, 20) = 22.79, *p* = 0.0001; duration: F­(4, 20) = 4.48, *p* = 0.0095) leaves.

After calculating the raw intensity values from the LFA strips, proportional values were determined, and normalized intensity values were subsequently computed using eq 2. Based on these analyses, LFA results for samples collected from healthy (uninfected) plants yielded normalized intensity values of 1.03 and 0.97 arbitrary units (au) for MN and swab sampling, respectively. For the reference *P. triticina* DNA concentrations of 0.4 ng/μL, 4 ng/μL, and 40 ng/μL, the corresponding normalized intensity values were 1.09, 1.63, and 1.95 (Figure 5B), confirming a concentration-dependent increase in LFA signal consistent with literature reports on NP-based hybridization assays.^36^ This trend is attributed to the higher amounts of amplified DNA generated at elevated input concentrations, which, in turn, increases the binding of DNA to the gold NP-conjugated probes on the LFA strip. This enhanced binding results in stronger color development at the test line (Figure S13). Thus, in subsequent experimental samplings using MNs and swabs, elevated normalized intensity values were indicative of greater DNA amplification, reflecting more effective sample collection and pathogen detection.

$$\mathrm{normalized}\;\mathrm{LFA}\;\mathrm{value}=\frac{\mathrm{test}/\mathrm{control}\;\mathrm{ratio}}{\mathrm{negative}\;\mathrm{control}\;\mathrm{results}}$$ 2

Pathogen detection efficiency was evaluated at multiple time points by using different sampling methods on both susceptible and resistant wheat leaves. While MN sampling enabled the collection of pathogens from both the plant surface and internal tissues, swab sampling was limited to surface-level pathogens. Sampling was conducted on days 1, 2, 3, 4, 5, and 15 post-inoculation. For each plant and time point, three MN-based samples were collected and averaged to represent the sampling efficiency (Figures S14–S25), whereas three swab samplings were performed once daily per plant. In susceptible leaves, MN-based sampling consistently yielded higher pathogen detection values than swabs at all time points (Figure 5C). For instance, on day 1, MN sampling resulted in an average detection value of 2.048 ± 0.059, compared to 1.688 ± 0.078 for swabs. This trend persisted through day 5 (MN: 2.084 ± 0.106, swab: 1.978 ± 0.091) and was maintained even on day 15 (MN: 2.312 ± 0.146, swab: 2.110 ± 0.175). Statistical analyses were performed using two-way ANOVA followed by Bonferroni’s multiple comparison test to evaluate the effects of sampling method (MN vs swab) and time point, and to determine whether specific differences existed between the groups. The analysis revealed that both duration (days 1–5) and sampling method had statistically significant effects on the results. Specifically, duration yielded a sum of squares (SS) of 0.3461 with 4 degrees of freedom (DF), a mean square (MS) of 0.08653, F­(4, 20) = 12.31, and *p* < 0.0001, while the sampling method showed an SS of 0.2730, DF = 1, MS = 0.2730, F­(1, 20) = 38.84, and *p* < 0.0001. Post hoc Bonferroni analysis indicated that day 1 (*p* = 0.0002) and day 3 (*p* = 0.0237) differed significantly from the other time points (*n* = 3) (Figure 5C).

Conversely, in resistant leaves, swab sampling generally outperformed MNs, pointing to a different infection dynamic (Figure 5D). On day 1, the swab yielded a detection value of 2.022 ± 0.155, higher than MN’s 1.759 ± 0.179. This trend continued until day 5, with values of 2.088 ± 0.1304 and 1.973 ± 0.091 for the swab and MN, respectively. The same statistical analyses were applied to the data collected from resistant leaves to determine whether any specific differences existed between the experimental groups. Two-way ANOVA revealed that both the duration (days 1–5) and the sampling method (MN vs swab) had statistically significant effects on the results. Specifically, duration exhibited an SS of 0.3440 with 4 degrees of freedom, an MS of 0.08601, F­(4, 20) = 4.480, and a *p*-value of 0.0095. The sampling method showed an SS of 0.4375, DF = 1, MS = 0.4375, F­(1, 20) = 22.79, and a p-value of 0.0001. Post hoc analysis using the Bonferroni multiple comparison test indicated that day 4 was significantly different from the other time points (*n* = 3, *p* = 0.0286) (Figure 5D). The data establish that in resistant leaves, intrinsic defense mechanisms inhibit internal pathogen penetration, indicating that surface sampling alone enables effective detection. In contrast, in susceptible leaves, MN-based sampling consistently produced higher detection values than swabs across all days. This persistent elevation reflects the progressive internal colonization of *P. triticina* within mesophyll tissues, which MNs can access but swabs cannot. Over time, as hyphal networks expand below the cuticle, the MNs continue to retrieve pathogen DNA from these subsurface regions, whereas swabs increasingly lose efficiency as surface spore concentrations plateau. The time-dependent divergence between sampling methods, also demonstrated in Figure S26, exhibits the progressive accumulation of pathogens within internal tissues of susceptible plants. The slight intensity variations observed between consecutive days may arise from minor differences in LFA visualizations and pathogen sampling efficiency; however, the consistent superiority of MN sampling across all time points confirms its suitability for detecting subsurface pathogens in susceptible plants. Overall, the outcomes establish that MN-based sampling enables reliable detection of internal infections in susceptible plants, while conventional swab sampling remains sufficient for identifying surface-localized pathogens in resistant plants. In addition to sampling tests for resistant and susceptible leaves, we also verified that our platform did not introduce assay artifacts. Specifically, we stated that while biological samples could inherently contain inhibitors (e.g., nucleases, ions, and secondary metabolites), our isolation-free MN-based strategy did not introduce additional inhibition from the 3D-printed material itself, as demonstrated by the control experiments (Figure S27).

To further validate MN sampling performance, additional sampling was conducted on day 15 using a portable diagnostic sensor (Figure 6A–D). The sensor’s results closely matched those of the laboratory-based system, with MN and swab values recorded at 2.307 a.u. ± 0.085 a.u. and 2.108 a.u. ± 0.137 a.u., respectively, which were not statistically significant according to the *t* test (Figure 6E–G), and this demonstrated the sensor’s reliability and suitability for field-based diagnostics (Figure S28). Since experiments were performed using two different heating systems, a bench incubator and a portable heater, minor variations between the results were expected. These differences likely arose from variations in device temperature control performance and the inherent variability of the LFA visualization. These results highlight the significance of choosing appropriate sampling strategies based on plant resistance profiles. MNs are optimal for susceptible plants due to their ability to access both surface-associated spores and internal pathogen reservoirs, while swabs are more efficient for resistant plants where pathogen ingress is blocked at the tissue level, and infections remain confined to the surface. Although the maximum difference between MN and swab readings was around 12% in susceptible leaves, this disparity is significant when interpreted in the context of the LFA signal behavior. For instance, in calibration studies, an ∼15% increase in T/C ratio corresponded to nearly a 10-fold change in template DNA concentration, underscoring that even moderate signal shifts can represent substantial biological differences. Therefore, rather than being negligible, such variations confirm the high sensitivity of MN-based sampling for detecting low pathogen loads. In addition to LFA tests, to prove the DNA amplification as a result of the LAMP test, samples were run on an agarose gel at various sampling points. Results showing that amplification of DNA was achieved as expected in a typical LAMP reaction, resulting in different lengths of DNA amplicons, which later formed a smear on agarose gel (Figure S29).

**Figure 6:**
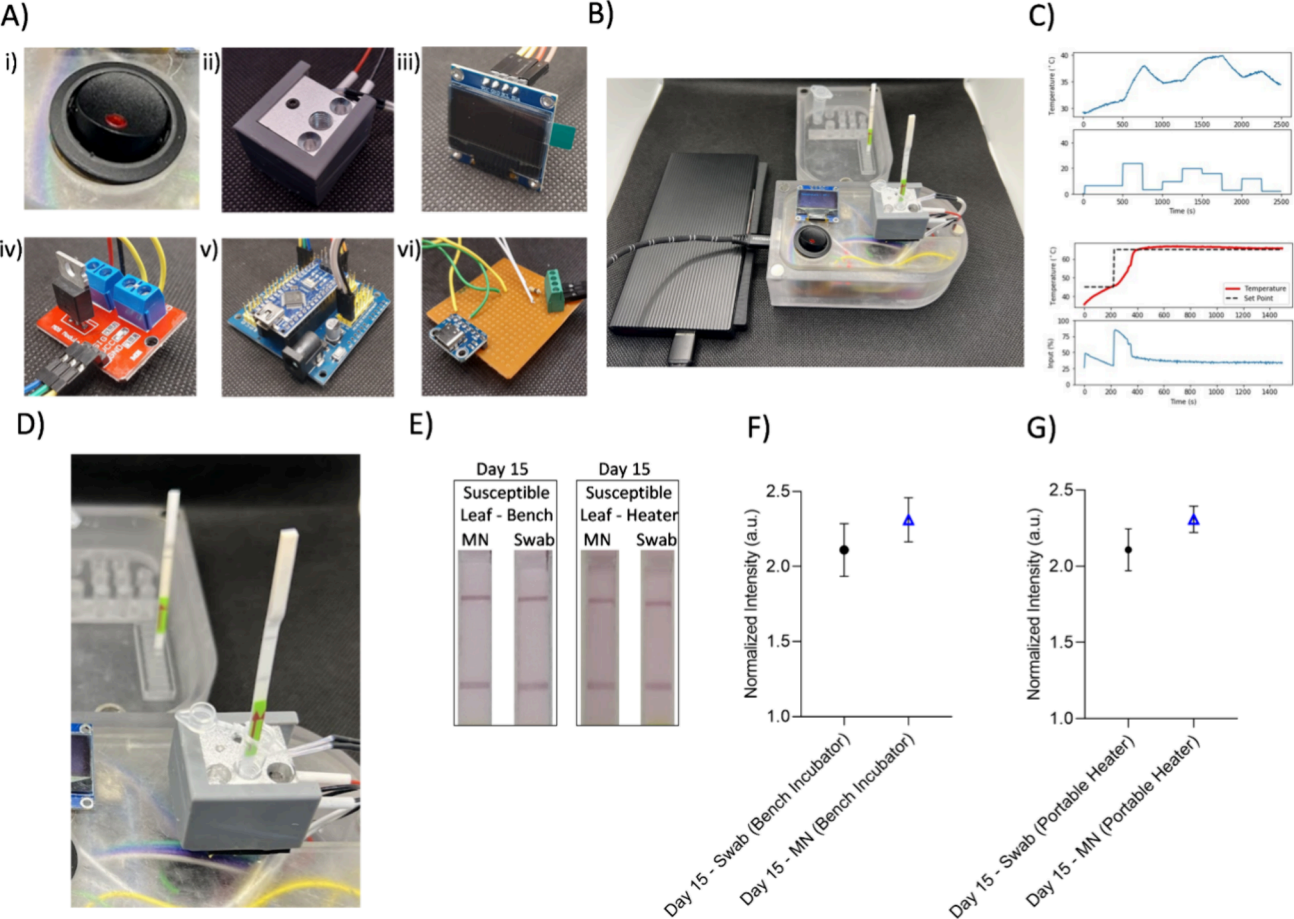
A portable heater, LAMP, and LFA were integrated to create a fully portable plant pathogen detection platform. (A) Individual components of the heater system. (i) ON/OFF switch. (ii) Custom heater unit made by drilling holes into a 3D printer heat block. (iii) LCD TFT screen for viewing the system temperature. (iv) MOSFET–Arduino interface board. (v) Arduino Nano on an adapter shield. (vi) Interface board for wire connections. (B) Fully assembled device running an LAMP test. The device is connected to a smartphone powerbank. (C) System identification (above) and PID Tuning (below) system temperature (below). (D) LAMP reactions were conducted using the portable heater, and LFA analysis was performed on the susceptible leaf sampled on day 15. (E) LAMP analyses for the 15th-day susceptible leaf were carried out using both a bench incubator and the portable heater to assess the efficiency of the portable heater, comparing LFA results from swab and MN sampling. (F) Image analyses of LFA results obtained from the bench incubator were performed, and no statistically significant difference was observed between the groups according to the *t* test (*n* = 3, *p* > 0.05). (G) LFA results from the portable heater showed comparable results to those obtained from the bench incubator according to the *t* test (*n* = 3, *p* > 0.05).

In summary, 3D-printed MNs outperformed PVA MNs in sampling efficiency and mechanical robustness, particularly for accessing internal tissues without structural degradation. While PVA MNs offer hydrophilicity-driven absorption, their partial dissolution restricts reuse and introduces variability in the sample volume. Conversely, cross-linked resin MNs provide structural rigidity, minimal deformation, and superior reusability under mechanical stress. Together with portable LAMP–LFA diagnostics, this integrated MN platform enables early, reliable detection of *P. triticina* under field conditions. Future work would refine the quantification accuracy through digital imaging or electrochemical readouts, further advancing on-site plant pathogen monitoring. Integrating real-time readout systems with digital image analysis or fluorescence NP probes could increase the detection sensitivity for low pathogen loads. Additionally, electrochemical or kinetic monitoring of signal development could further differentiate sampling efficiencies by quantifying early colorimetric responses, ultimately providing a higher-resolution understanding of pathogen dynamics throughout infection progression.

## Conclusions

This study presents two major contributions: (i) a comparative evaluation of different MN types for plant pathogen sampling and (ii) the development of a sensitive, field-deployable sensor system for *in situ* pathogen detection. Hydrophilic PVA-based MNs, widely used for plant tissue sampling, demonstrated limitations in our experiments due to their solubility during extraction, which could compromise the integrity and accuracy of downstream diagnostics. In contrast, 3D-printed MNs maintained their structural integrity during sampling, resulting in a superior extraction efficiency. While our observations confirmed that these MNs possessed high mechanical durability, in this study, each MN was used only once per sampling to eliminate potential cross-contamination issue. Their mechanical robustness also offers significant economic and environmental advantages, positioning them as a sustainable alternative to single-use PVA MNs. In particular, our results demonstrated that 3D-printed MNs are more effective at sampling pathogens both on the surface and within the internal tissue of infected plant leaves. Moreover, these MNs enabled consistent disease detection over a 15 day period, underscoring their robustness for longitudinal monitoring. Looking ahead, in terms of material selection, alternative material strategies such as swelling hydrogels or composites may further enhance analyte uptake while maintaining mechanical integrity, representing a promising direction to combine efficiency with stability.

One of the key findings demonstrated in this study is that modifying the printing angle markedly enhances tip sharpness, with a 75° orientation producing tips about 27.12% sharper than those printed at lower angles. Regarding dimensional accuracy, titanium MNs produced by EDM exhibited less than 1% shrinkage in height. Similarly, PVA MNs showed an approximately 5% shrinkage. In contrast, 3D-printed MNs exhibited around 10% shrinkage due to the limitations in printer resolution and material contraction during postcuring. Even with these shrinkage limitations, SLA-based 3D printing remains highly advantageous for plant-oriented applications. Its manufacturing flexibility allows for rapid adaptation of MN arrays to different plant species by modifying tip sharpness, needle density, and overall patch dimensions. This versatility makes it possible to match the device’s geometry to the varying thicknesses and structures of various leaves, thereby improving penetration efficiency and sample recovery without the need for extensive retooling or mold fabrication. In future studies, smaller patches or MNs with reduced heights (e.g., 50–75 μm) tailored to specific plant geometries, such as narrow leaves or sap-rich tissues, would be explored. However, the fabrication of such shorter MNs is currently constrained by the resolution limits of the 3D printing systems. In parallel, autonomous or robotic sampling platforms could be potentially developed to achieve highly precise, tissue-specific penetration, representing a complementary direction for future research.

The second major pillar of this study involved the successful field deployment of a portable diagnostic system. Pathogen DNA was amplified using in-field LAMP reactions, and detection was achieved using LFA strips. The performance of this $25 portable system was on par with that of conventional benchtop incubators, confirming its potential for accurate, on-site diagnostics. Here, our system successfully determined infected plants with a 0.4 ng/μL DNA of detection limit on the LFA assay, operated up to 1–2 ng/μL DNA extraction per MN sample. Importantly, this amplification strategy did not rely on a separate nucleic acid extraction step, as direct addition of primary plant samples to the LAMP mix yielded a sufficient template for amplification. Since the MN-based sampling mechanism is nonspecific, host plant DNA is inevitably coextracted together with pathogen DNA. While this did not interfere with pathogen-specific amplification due to the high selectivity of the LAMP primers, this feature would be leveraged in future studies to design multiplex assays capable of simultaneously identifying the host plant species and detecting associated pathogens. In addition, while this simplifies field deployment by eliminating additional reagents and equipment, future implementations may incorporate LAMP-compatible extraction buffers or simple preheating steps to further improve the detection limit. This capability enables rapid, laboratory-independent detection in agricultural environments, such as fields and greenhouses, significantly accelerating the disease response time and improving the efficiency of agricultural management practices.

Despite these advancements, a potential limitations persist. The mechanical performance of SLA-printed MNs remains dependent on the resin formulation, necessitating further optimization to enhance the absorption and durability. Additionally, the integration of microsensors or artificial-intelligence-supported analysis platforms could automate pathogen identification and facilitate the creation of real-time databases, paving the way for data-driven disease management systems. Future studies would also investigate optimal and field-adapted washing and sterilization protocols to enable safe reuse of 3D-printed MNs. In this context, developing a portable platform, such as a compact in-field stirrer system, for on-site decontamination could further enhance their sustainability and practicality. Importantly, our study provides a more comprehensive assessment of MN design parameters, particularly geometry and material composition, compared with prior literature, which often focuses on isolated material properties or standardized geometries. By highlighting the synergistic effect of multiple design variables on mechanical and functional performance, this research lays the groundwork for future applications of 3D-printed MNs in the agricultural domain.

## Supplementary Material



## Notes

The detailed MN designs; the demonstration of MN application on plants; mechanical testing results including curing time optimization; resin material inhibition test on LAMP; LFA design and results; 10-cycle compression test; LAMPbox prototype design; image processing data (ImageJ) of LFA results; pathogen inoculation and lifecycle in plants with microscopy images; and agarose gel electrophoresis of pathogen sampling (PDF)
