# Supplementary material for: In-Field Detection of Plant Pathogens Using Three-Dimensional-Printed Microneedles and a Portable Platform
Source: ACS Sens. 2025 Nov 27;10(12):9397–410. doi: 10.1021/acssensors.5c02361 (PMC12751100; doi:10.1021/acssensors.5c02361)
Supplement: Supplementary file 1 [file se5c02361_si_001.pdf]

Supporting Information

In-Field Detection of Plant Pathogens Using Three-Dimensional-Printed Microneedles and a Portable Platform

Emre Ece[1,2,#], Nedim Hacıosmanoğlu[1,2,#], Murat Alp Güngen[1,2], Metin Burak Tatlıses[3], İsmail Eş[1], Semra Hasançebi[3,*], Fatih Inci[1,2,*]

[1] UNAM-National Nanotechnology Research Center, Bilkent University, 06800, Ankara, Turkey

[2] Institute of Materials Science and Nanotechnology, Bilkent University, 06800, Ankara, Turkey

[3] Department of Genetics and Bioengineering, Engineering Faculty, Trakya University, Ahmet Karadeniz Yerleskesi, 22030, Edirne, Turkey

[#] Contributed equally

[*] Co-corresponding Authors: finci@bilkent.edu.tr ; semrahasancebi@trakya.edu.tr

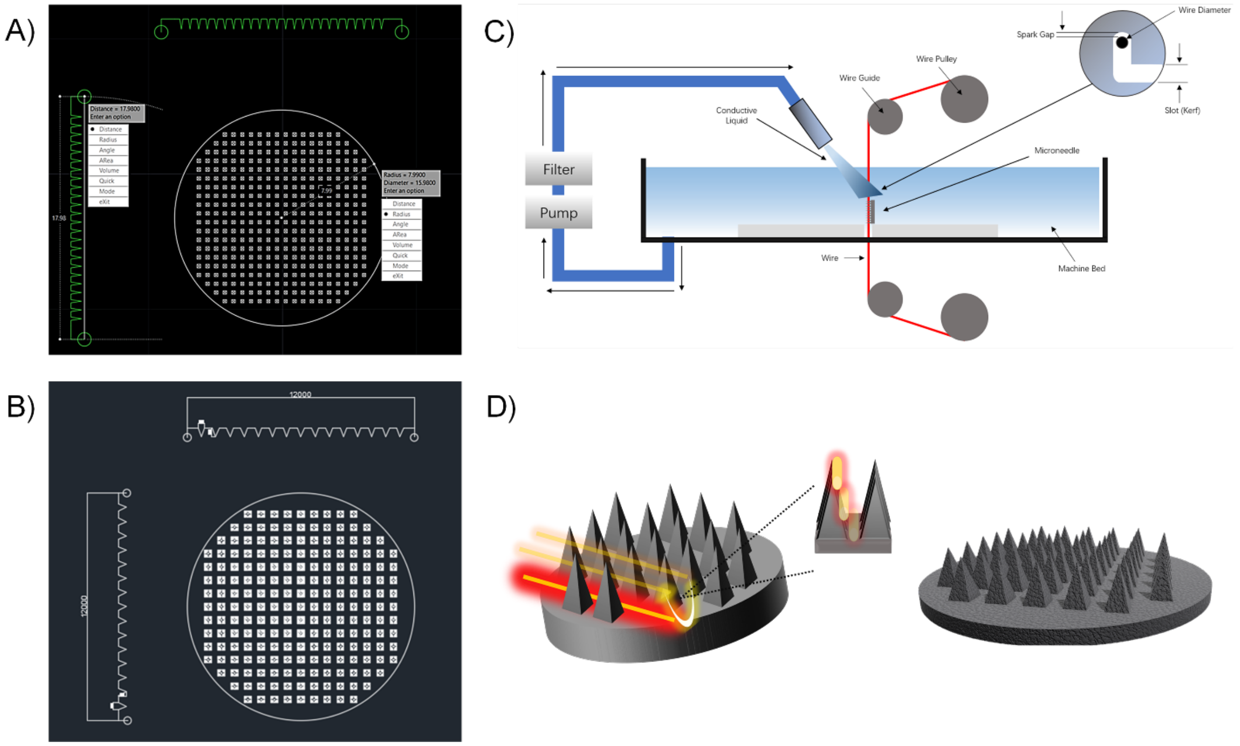

Figure S1. AutoCAD designs of (A) large titanium and (B) small titanium microneedles (MNs) were demonstrated with geometrical features. C) Working principle and main components of EDM was illustrated. D) The effect of hot wire on MN shaping (left) and the final titanium MN product (right) were exhibited.

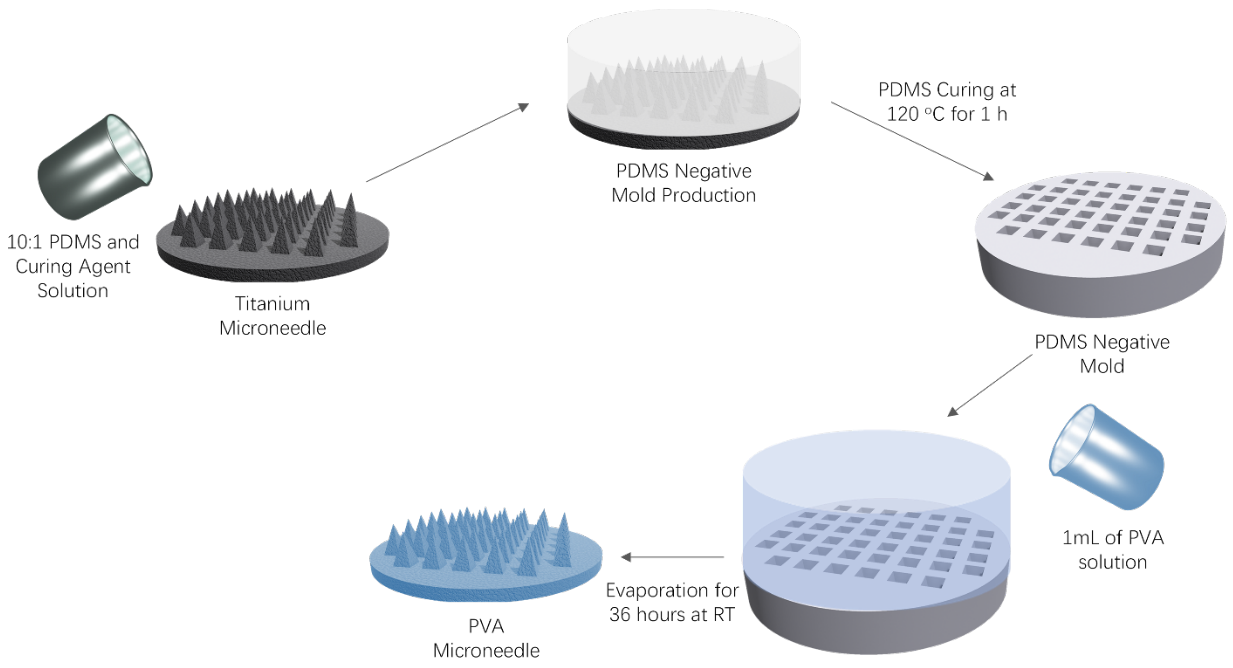

Figure S2. The workflow for fabricating PVA MNs, starting from PDMS negative mold production using a titanium MN and ending with solution evaporation, was illustrated.

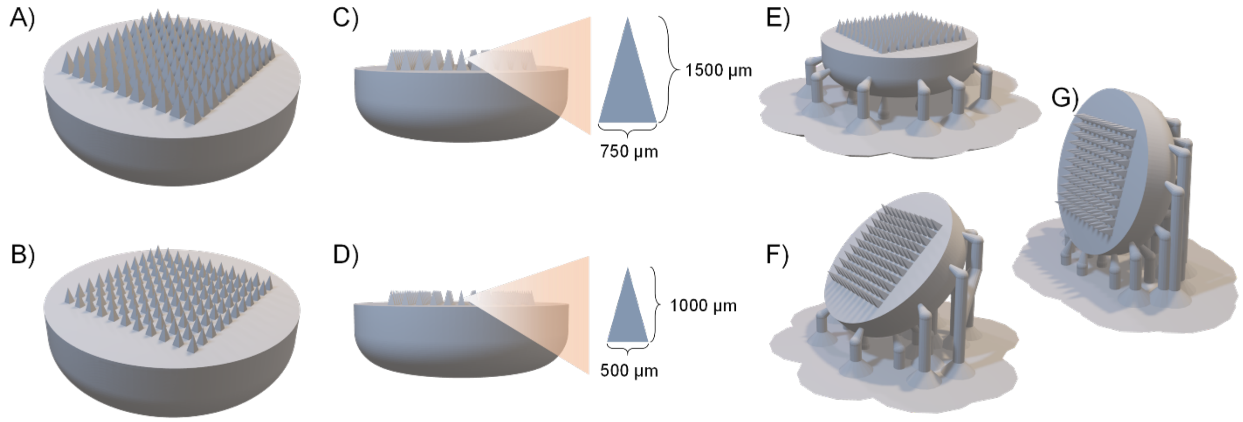

Figure S3. Designs of the 3D-printed MNs were demonstrated. A) A large 3D-printed MN and B) a small 3D-printed MN were shown. C) The geometric dimensions of the large MN, including height and width, were displayed. D) Height and width properties of the small MN were illustrated. E), F), and G) depicted printing angles of 0°, 45°, and 75°, respectively, along with their corresponding raft structures.

Table S1: PCR Primers of *Oryza sativa*

Species	Primer	Sequence 5′–3′	Product Size
Oryza sativa	RM259-F	TGGAGTTTGAGAGGAGGG	172-178 bp
	RM259-R	CTTGTTGCATGGTGCCATGT	

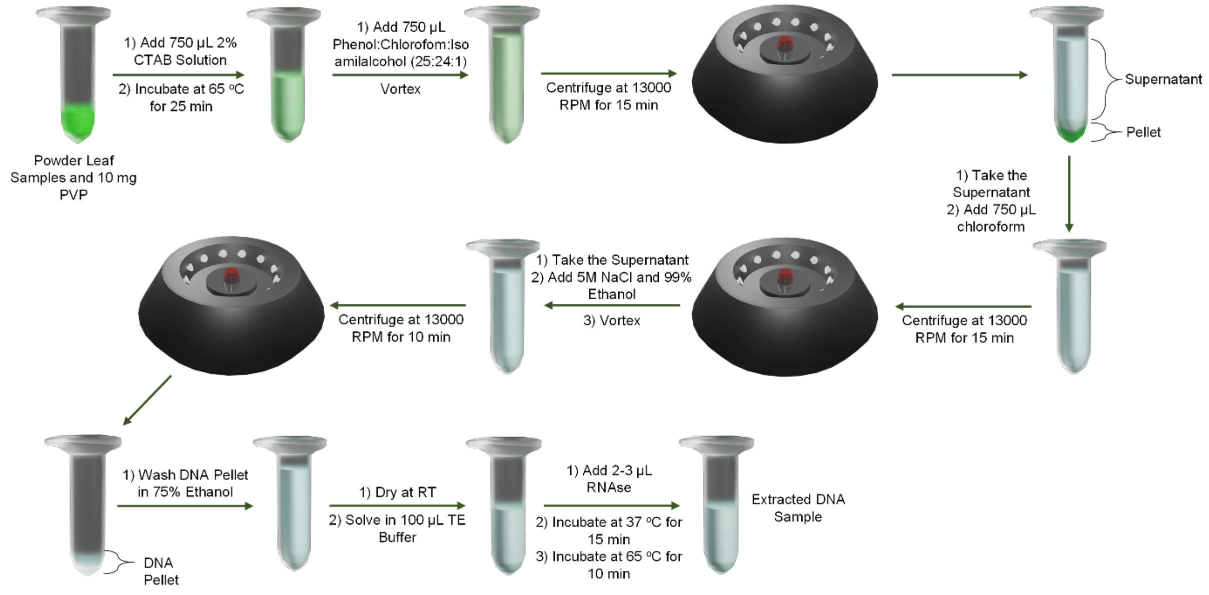

Figure S4. The schematic representation of the CTAB method's experimental setup was illustrated.

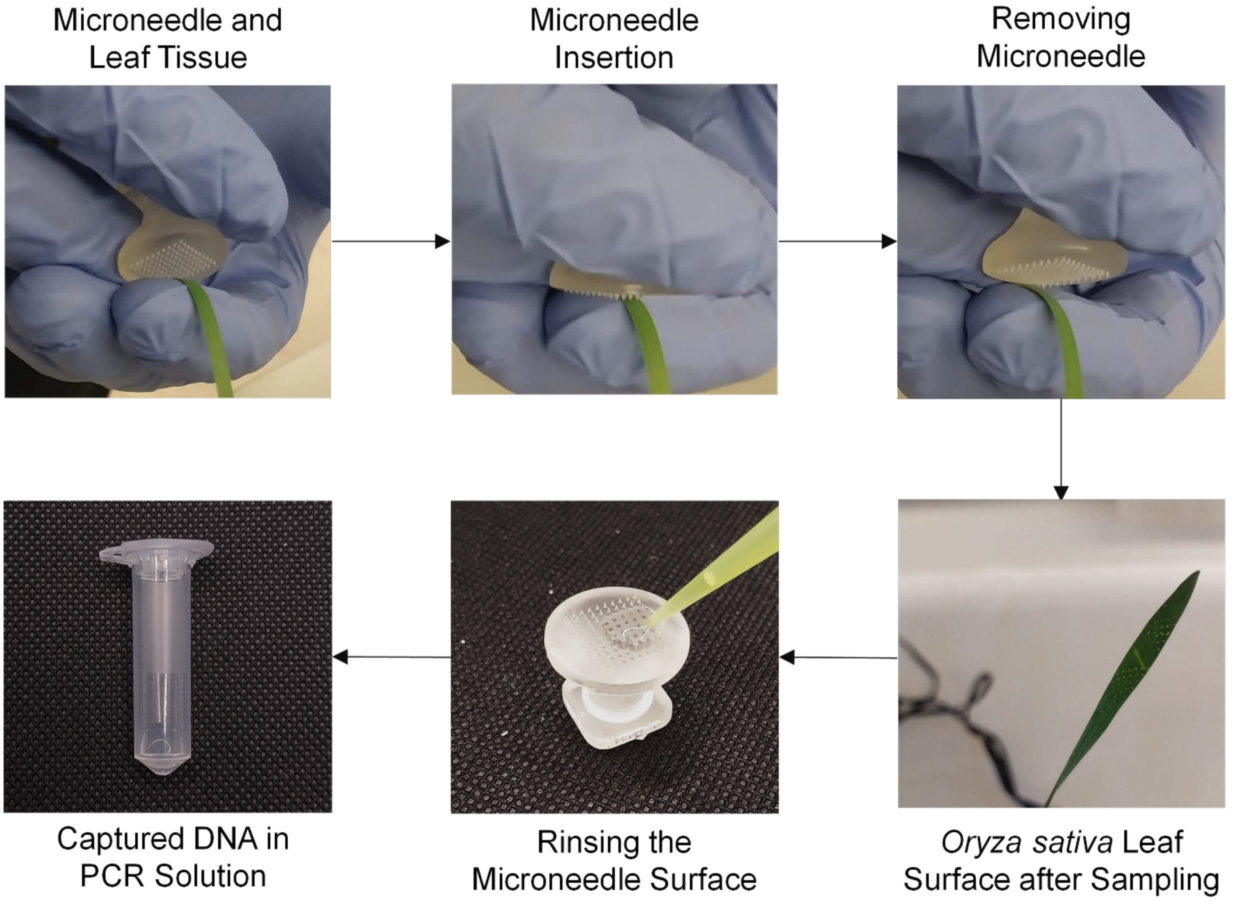

Figure S5. The experimental setup for DNA sampling using a 3D-printed MN was demonstrated through optical images.

Table S2: LAMP Primers of *P. triticina*

Primers	Modification	Sequence 5′–3′	bp
Pt-LF	FAM	GCTTGTCGGTGACGTGGTAG	20
Pt-LB	-	GACCAAATACGTCGCCATCGAG	22
Pt-F3	-	CACACACACACACACAGGT	19
Pt-B3	-	CGAGTAGAGCCGGATGCA	18
Pt-FIP	Biotin	GCGAACTGCTTGTTGGGGGGttttGGGATCCTACTCGACCGT	42
Pt-BIP	-	GTGCTCGACAAGCGCCACATttttTCGAGCAGGTTGAGGGTG	42

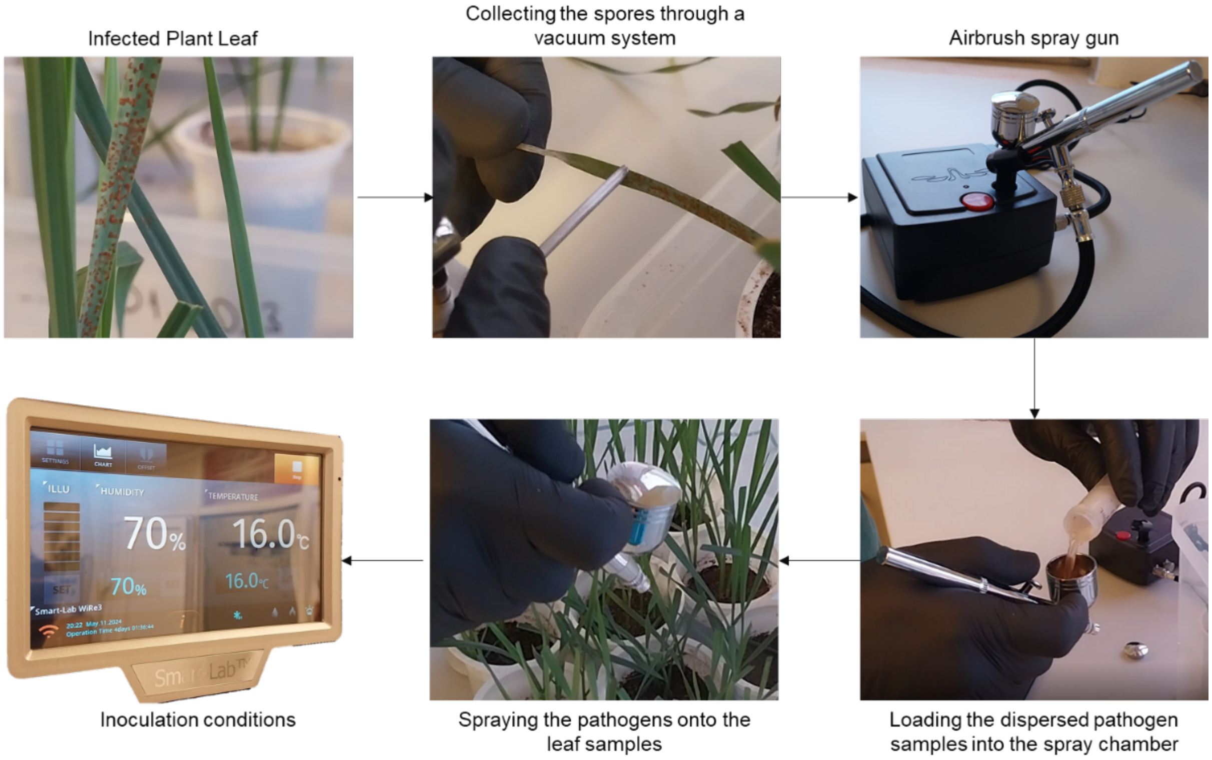

Figure S6. Optical images were used to illustrate the procedures for plant pathogen collection and inoculation.

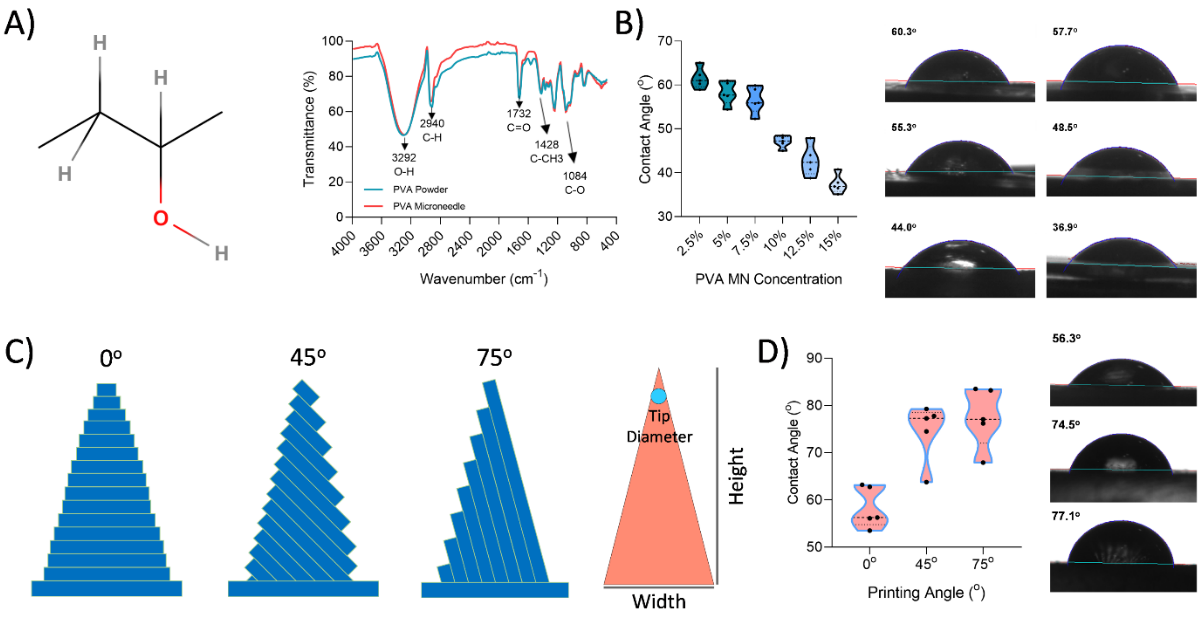

Figure S7. PVA and 3D-printed MNs were chemically and morphologically characterized. A) The PVA MN was chemically characterized using ATR-FTIR to analyze its functional groups, with a comparison made to the powdered form of PVA to verify any structural changes after MN fabrication. B) Contact angle measurements were conducted to investigate the effect of PVA concentration on hydrophilicity. C) The printing angles of 3D-printed MNs were illustrated, showing their corresponding geometrical properties. D) Contact angle measurements were repeated to evaluate the effect of printing angles on the hydrophilicity of the 3D-printed MNs.

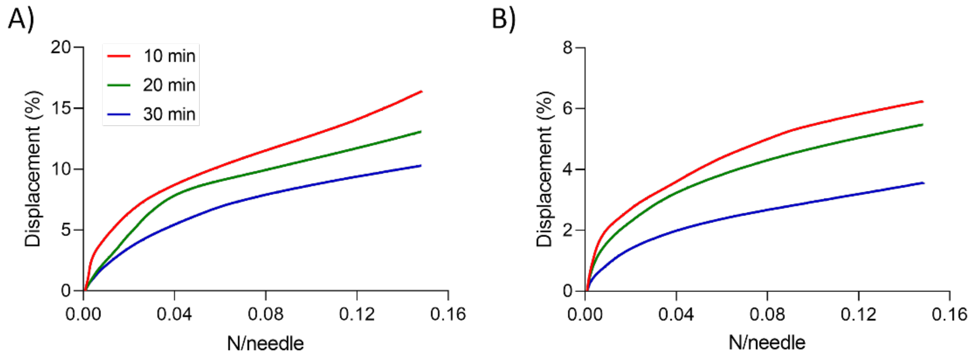

Figure S8. The effect of curing time on the mechanical properties of small (A) and large (B) 3D-printed MNs with a 0° printing angle was investigated.

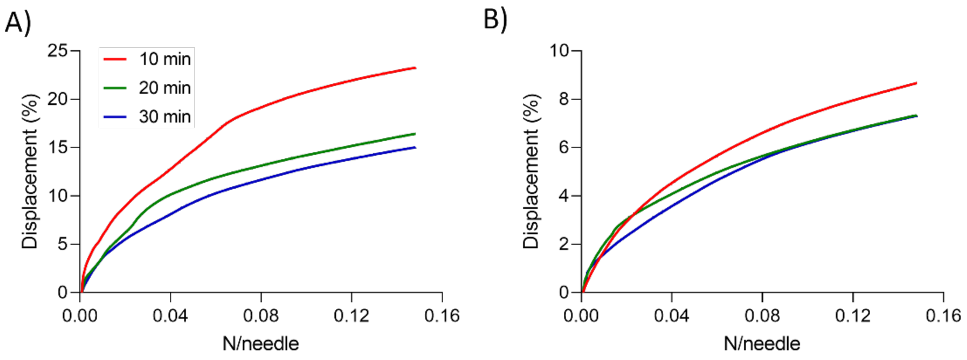

Figure S9. The impact of curing time on the mechanical properties of small (A) and large (B) 3D-printed MNs with a 45° printing angle was examined.

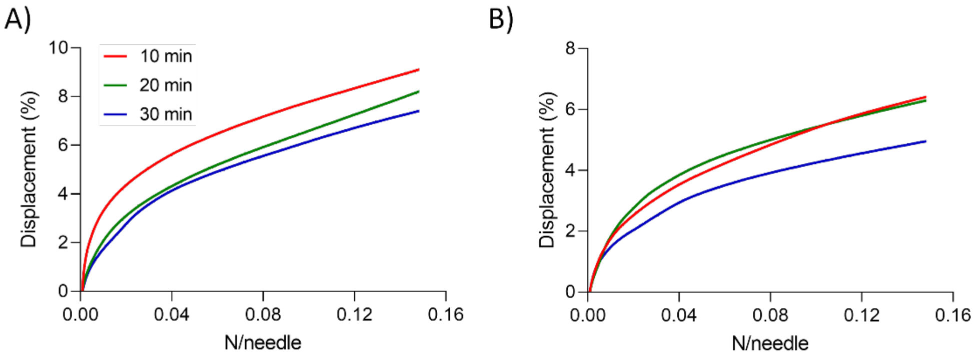

Figure S10. The impact of curing time on the mechanical properties of small (A) and large (B) 3D-printed MNs with a 75° printing angle was examined.

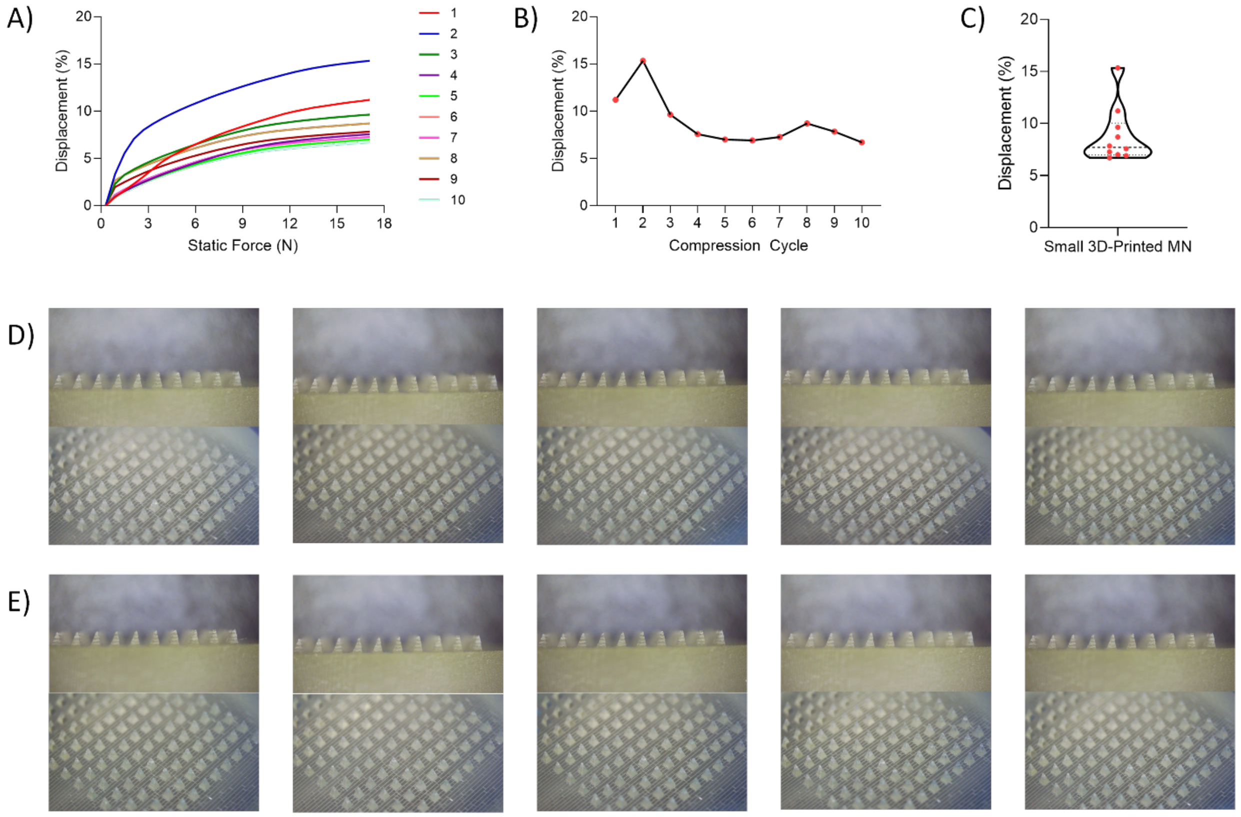

Figure S11: (A) Compression–displacement curve of the small 3D-printed MN. (B) Repeated compression cycles (n = 10) applied at 18 N/min, showing minor displacement fluctuations without cumulative structural failure. (C) Quantification of displacement variation over 10 cycles. (D–E) Optical microscope images of the same MN patch during repeated compression cycles, confirming structural integrity without visible tip damage (D: cycles 1–5; E: cycles 6–10).

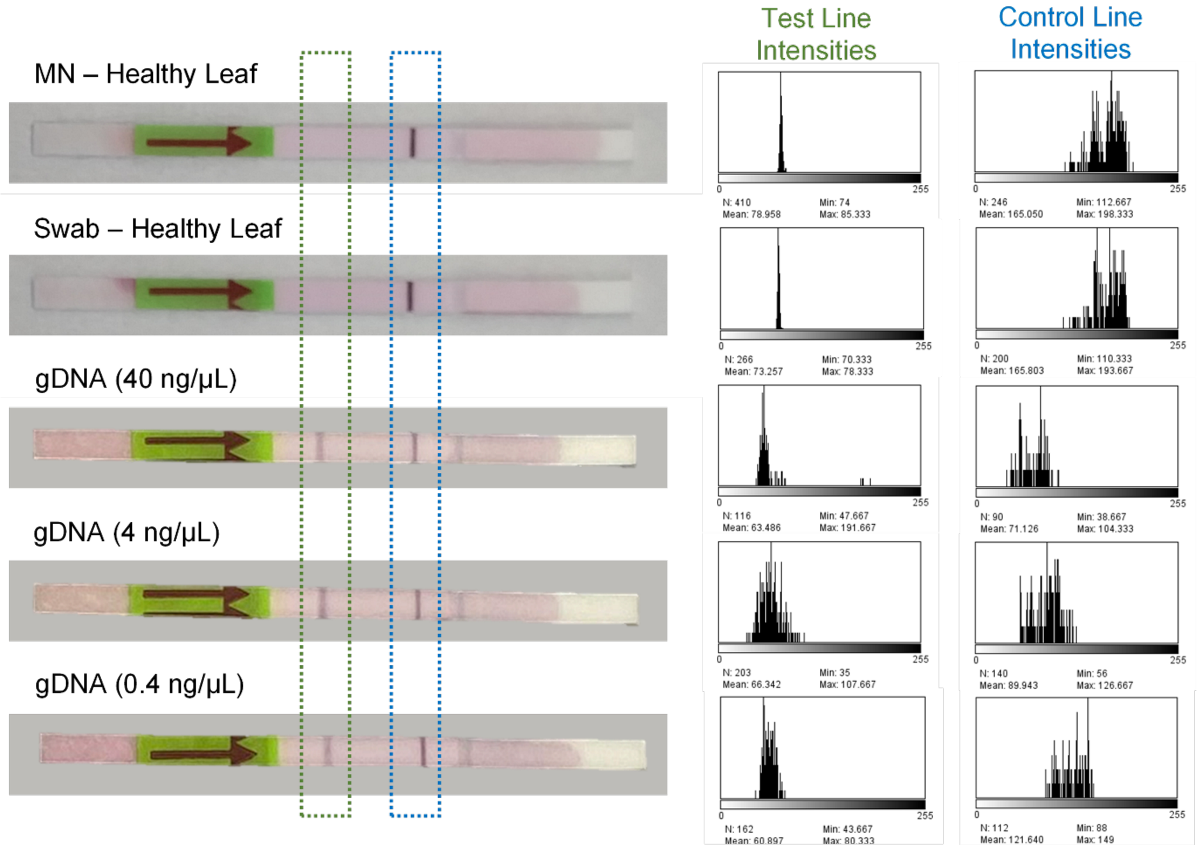

Figure S12. The results of LFA tests for healthy leaves and gDNA solutions, along with the intensities of their test and control lines, were presented.

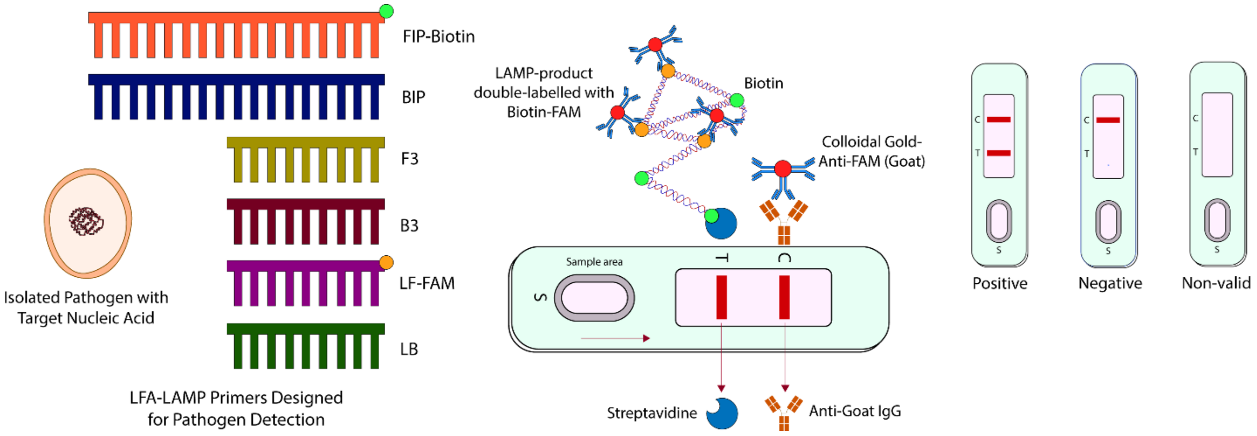

Figure S13. The design of the LFA for pathogen detection was illustrated.

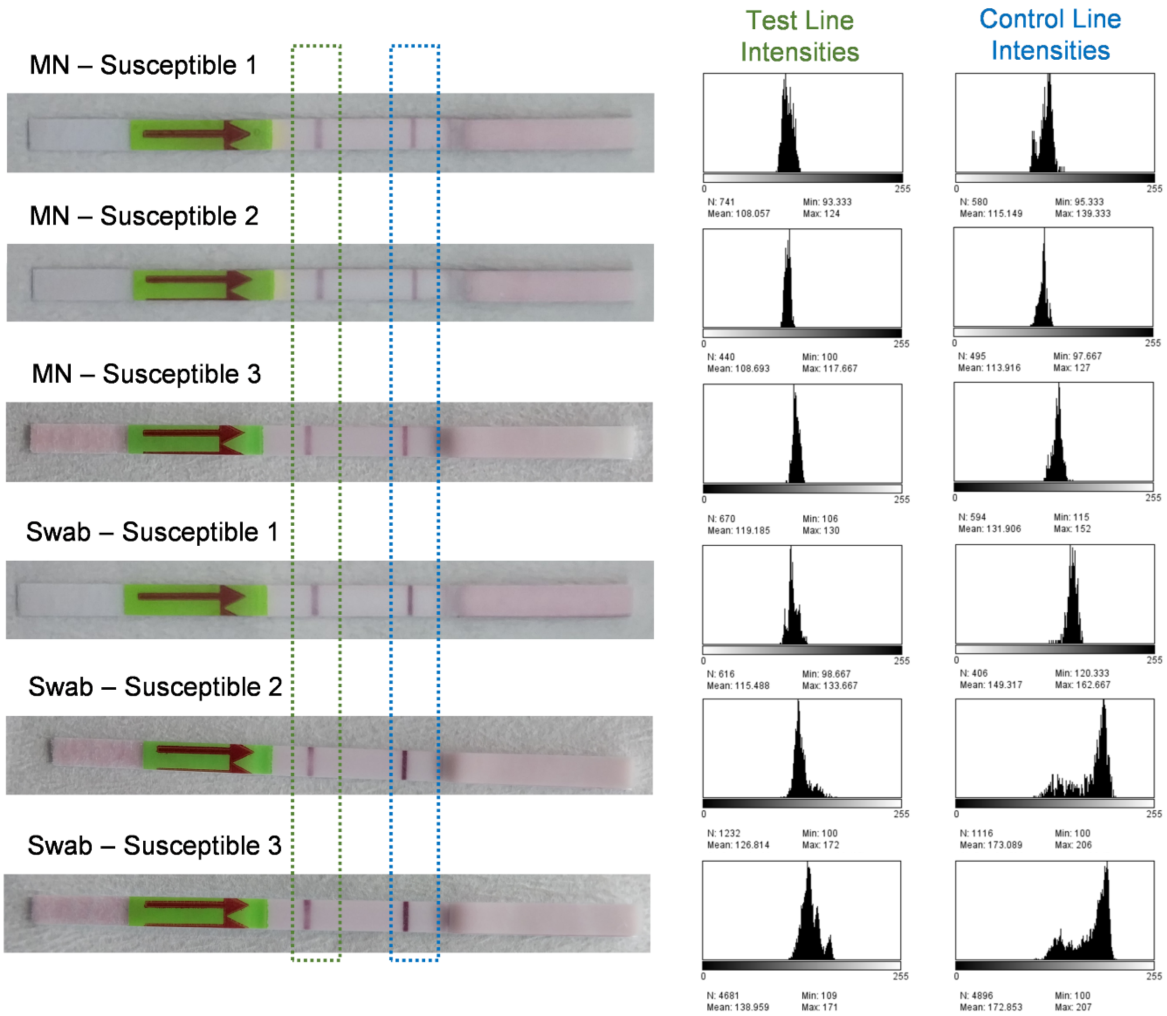

Figure S14. On the first day after inoculation, LFA test results and intensity values for both swab and MN samples from susceptible leaves were shown.

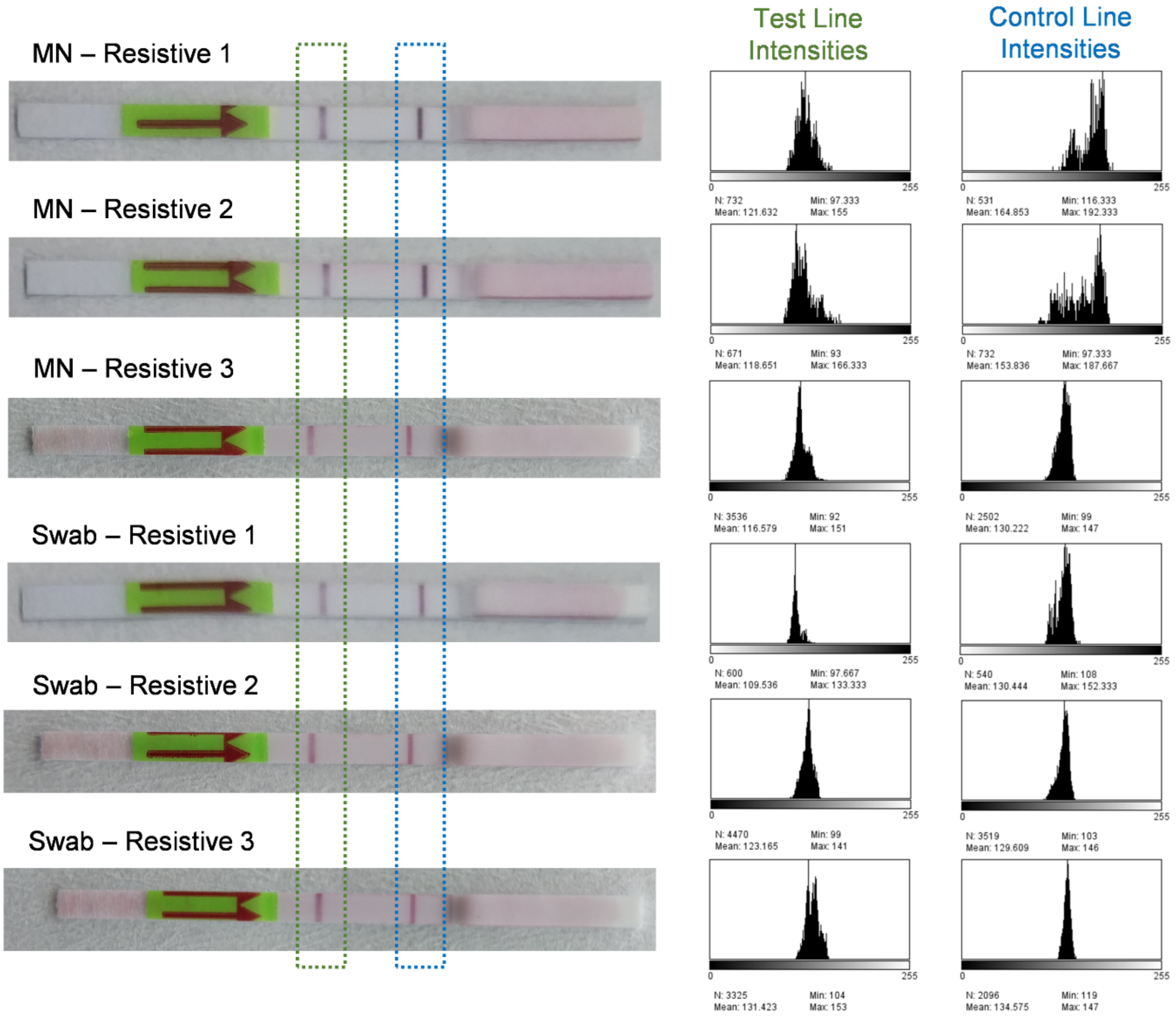

Figure S15. On the first day after inoculation, LFA test results and intensity values for both swab and MN samples from resistant leaves were shown.

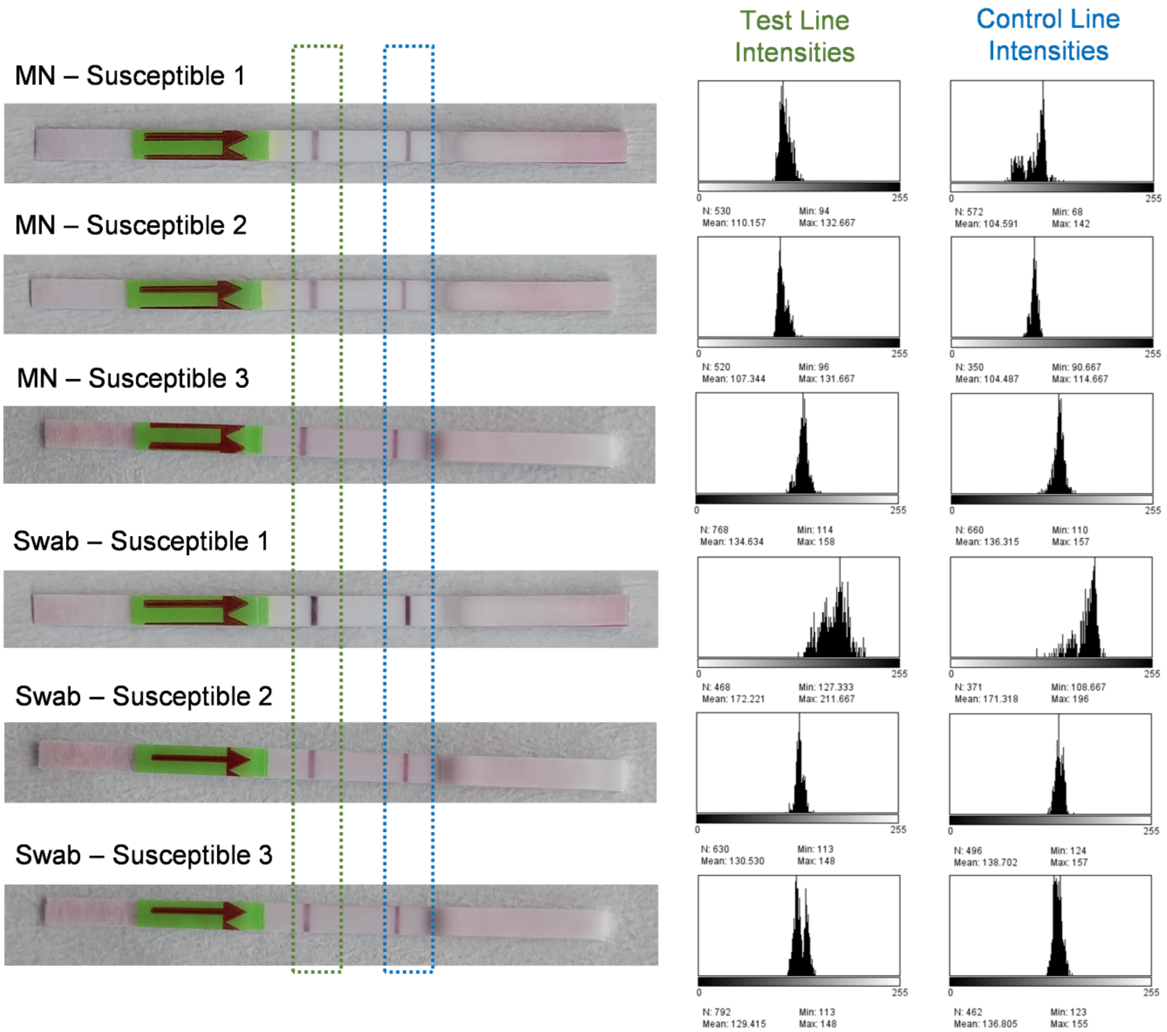

Figure S16. LFA test results and intensity measurements for swab and MN samples from susceptible leaves were recorded on the second day after inoculation.

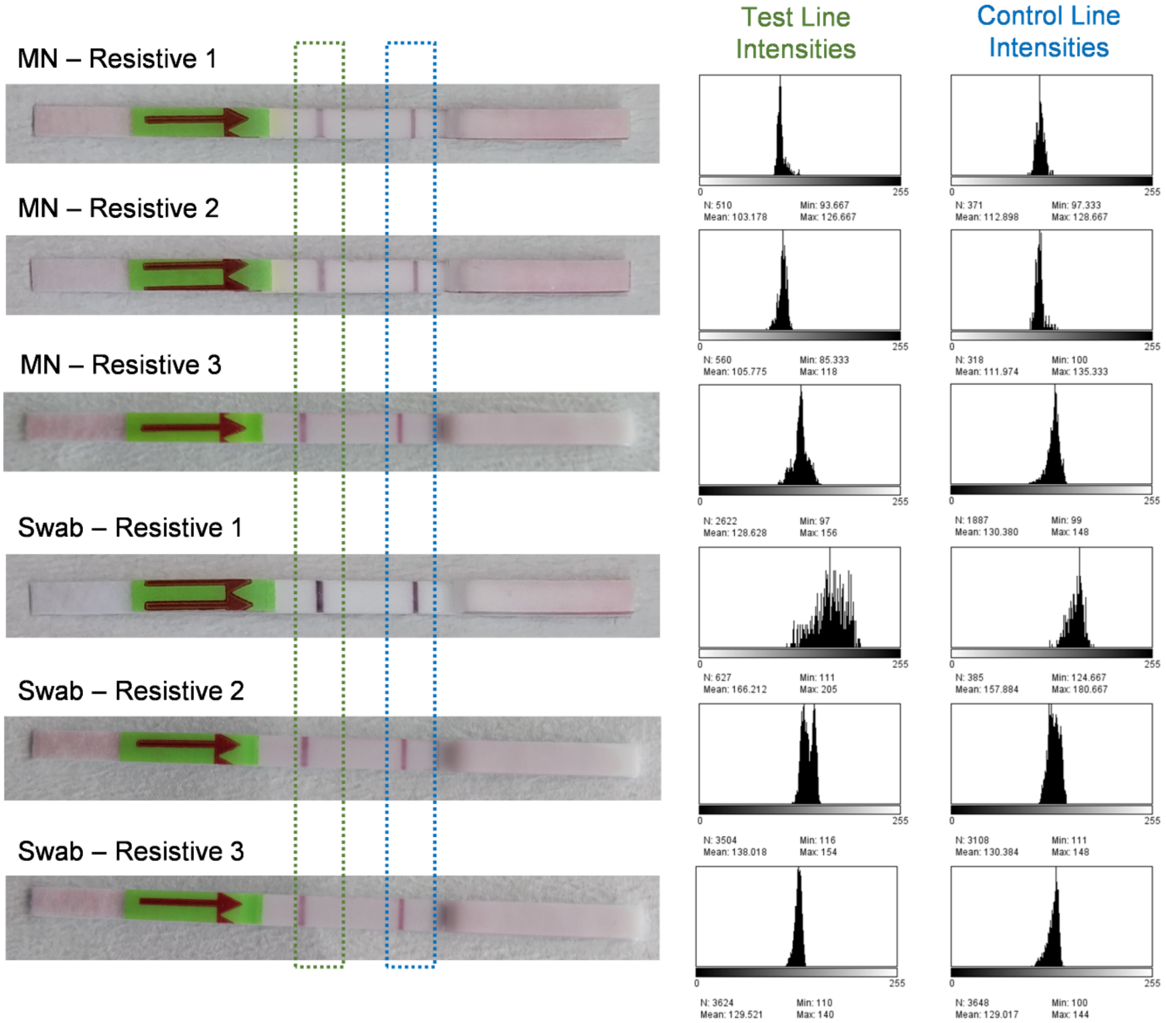

Figure S17. LFA test results and intensity measurements for swab and MN samples from resistant leaves were recorded on the second day after inoculation.

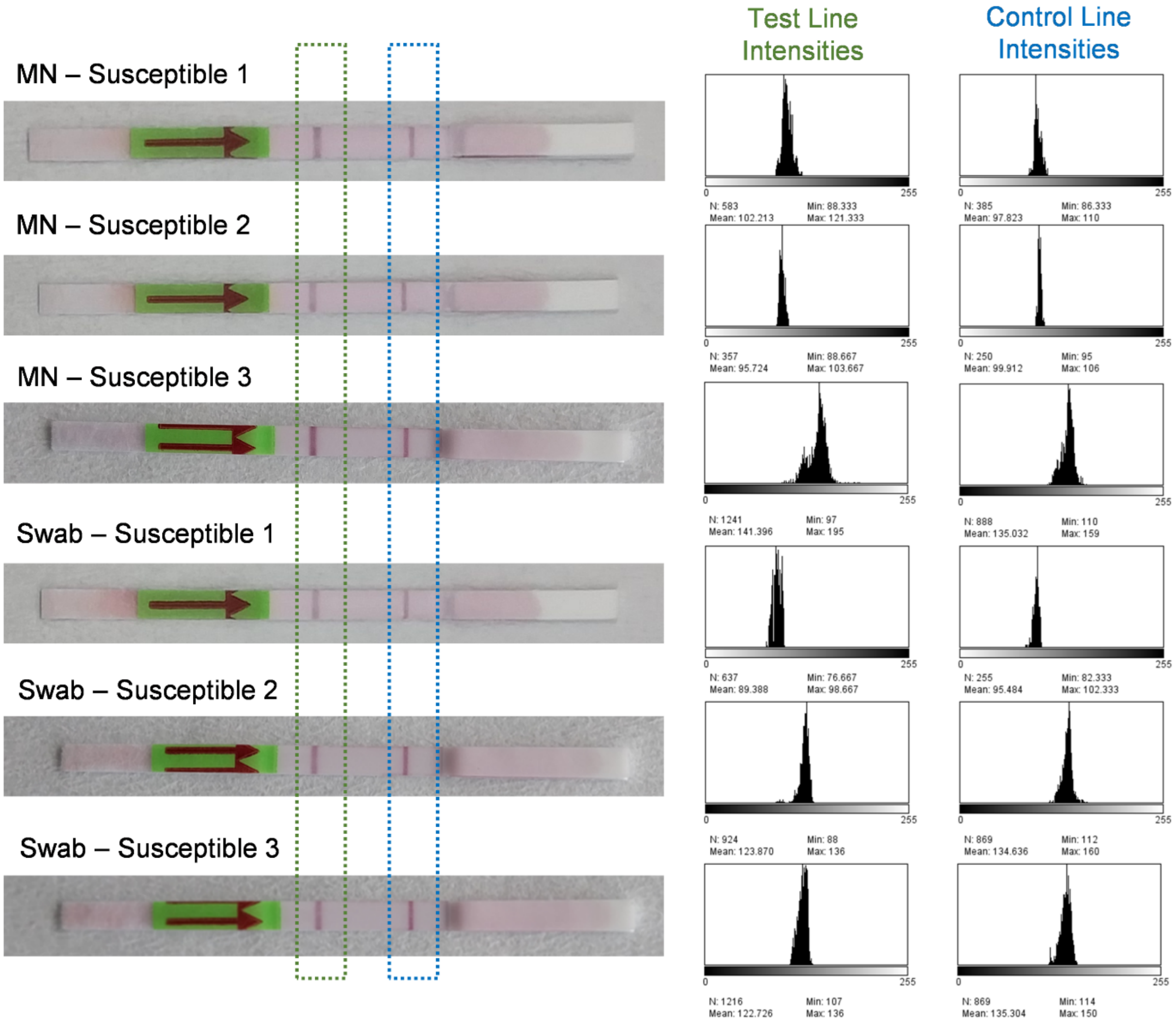

Figure S18. On the third day post-inoculation, intensity values from LFA tests on swab and MN samples collected from susceptible leaves were analyzed.

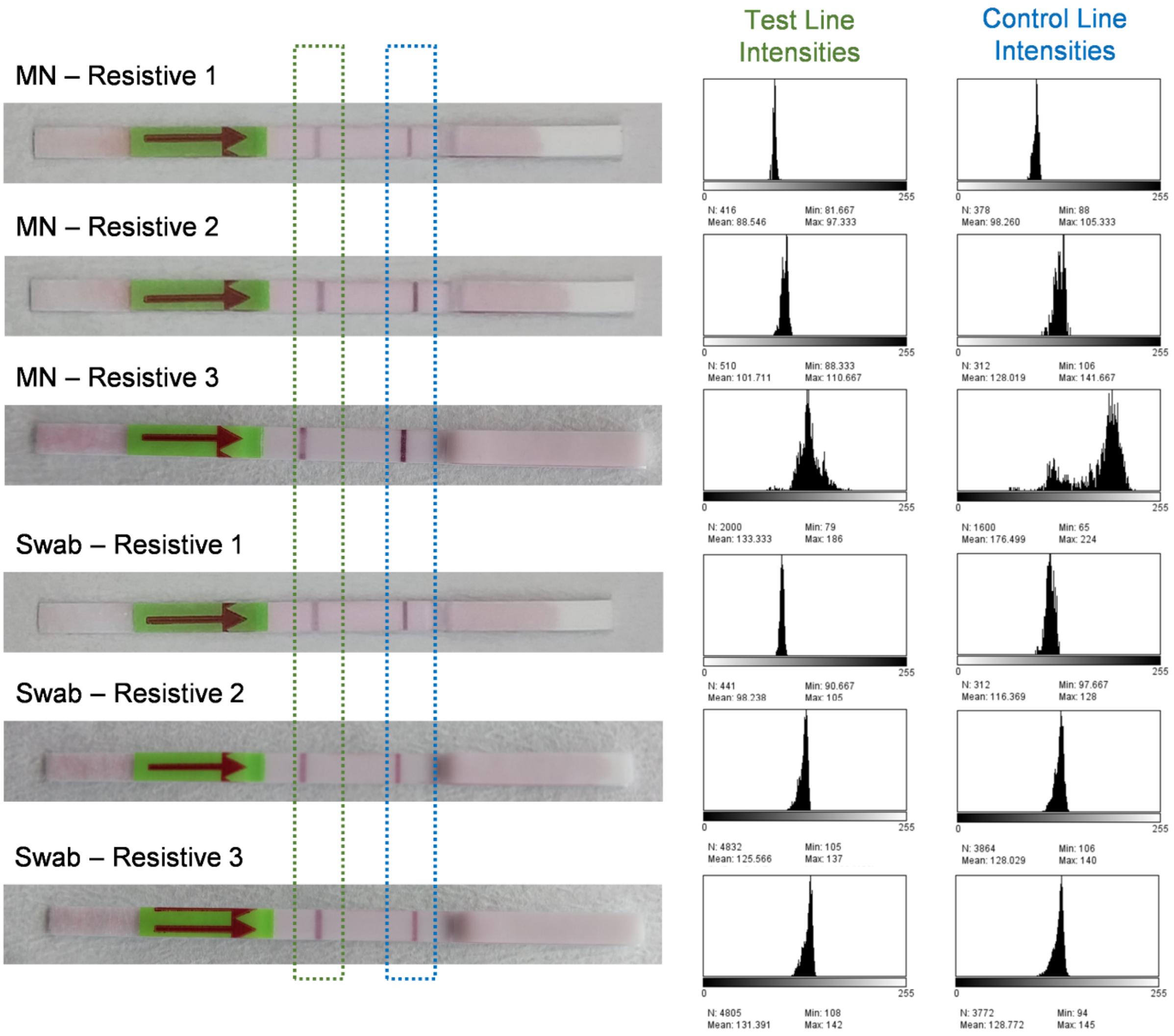

Figure S19. On the third day post-inoculation, intensity values from LFA tests on swab and MN samples collected from resistant leaves were analyzed.

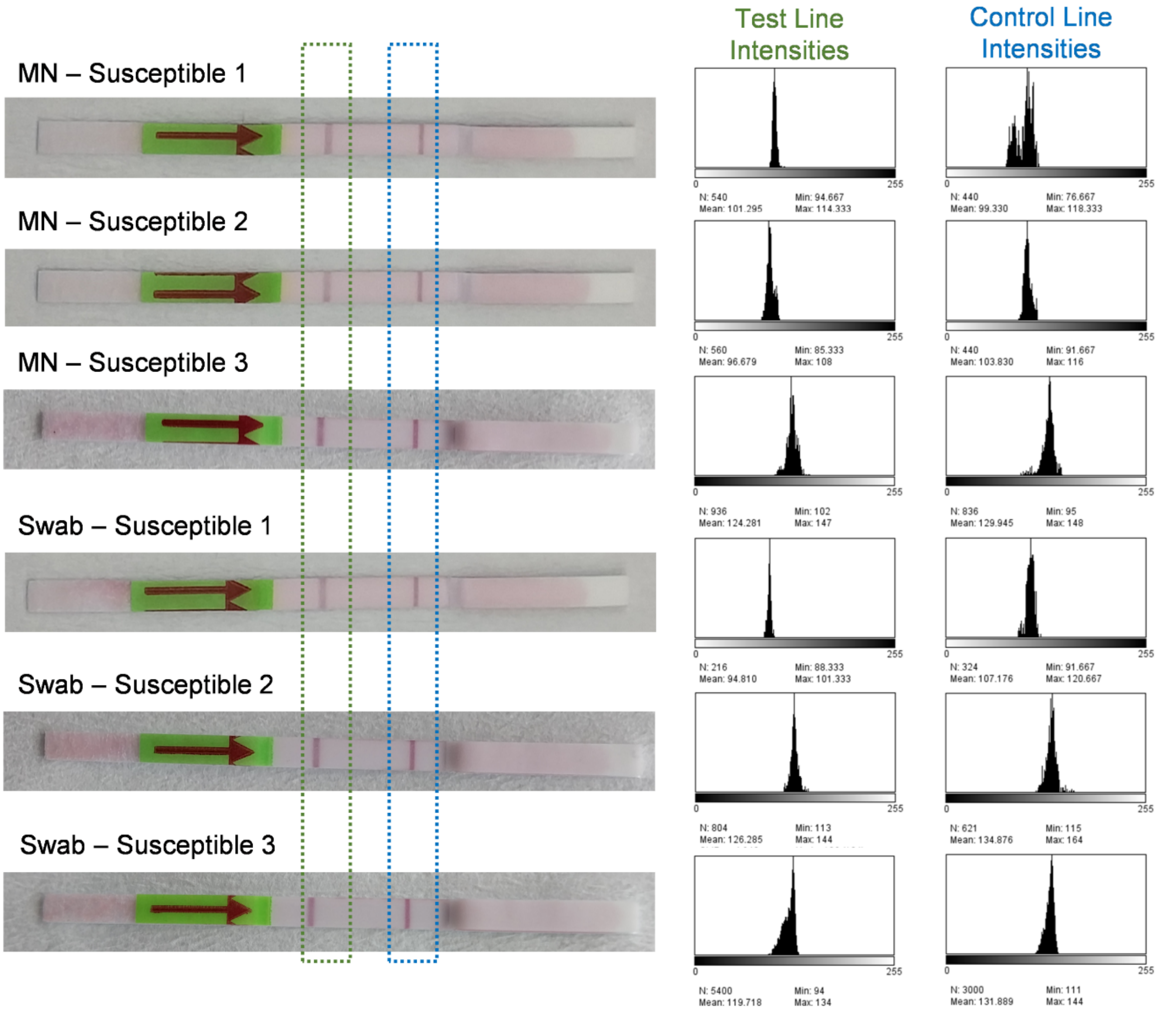

Figure S20. Intensity values from LFA tests conducted on swab and MN samples from susceptible leaves were examined on day 4 after inoculation.

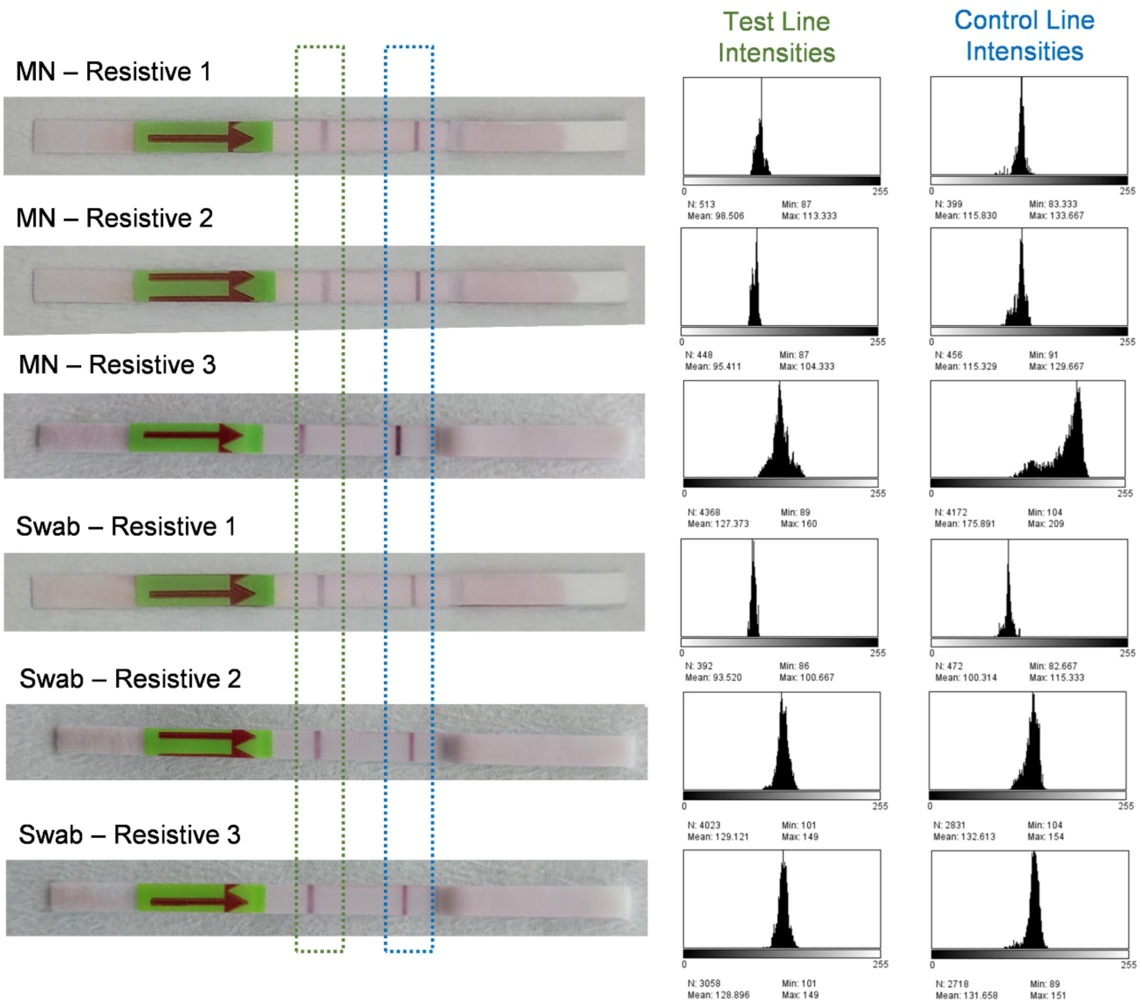

Figure S21. Intensity values from LFA tests conducted on swab and MN samples from resistant leaves were examined on day 4 after inoculation.

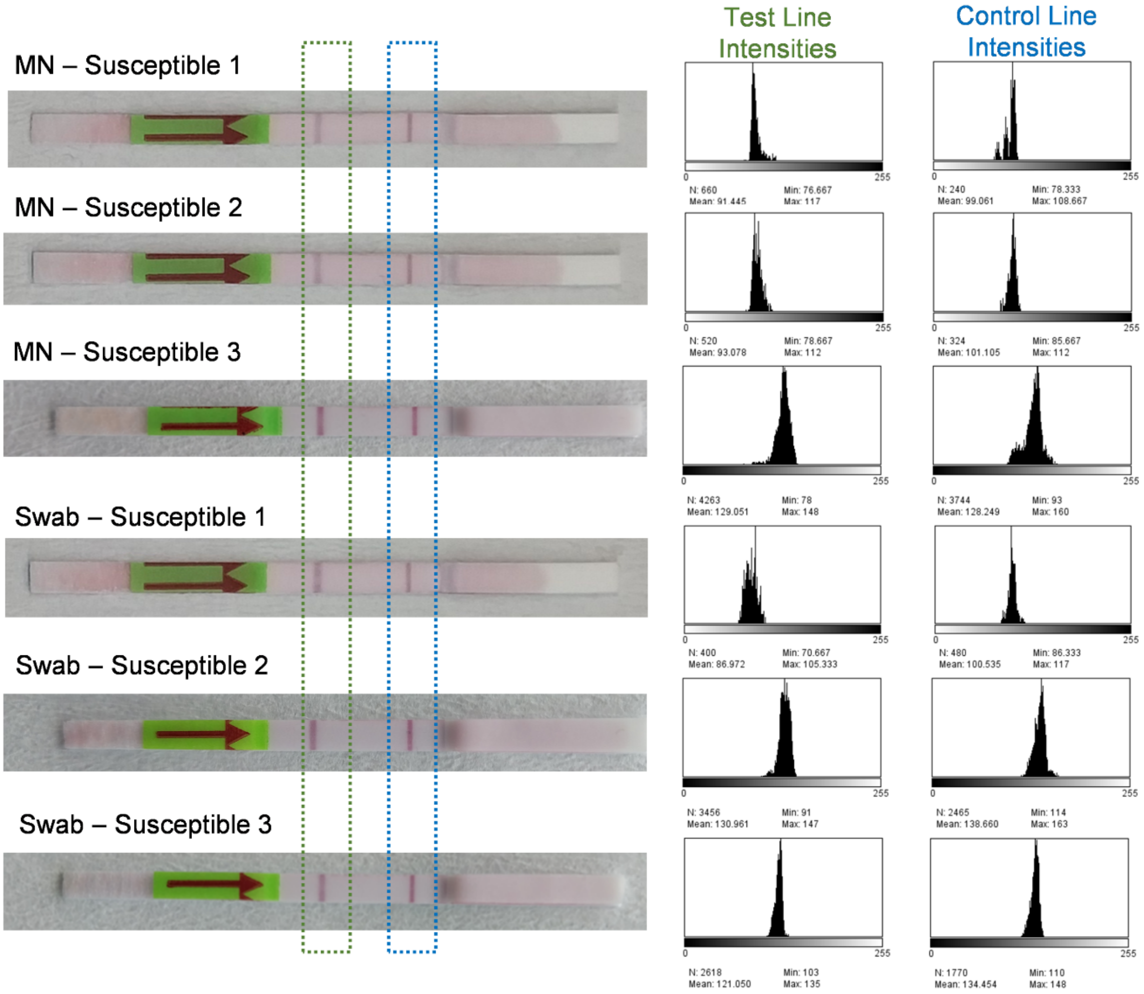

Figure S22. On the fifth day following inoculation, LFA test results for swab and MN samples from susceptible leaves, including their intensity values, were presented.

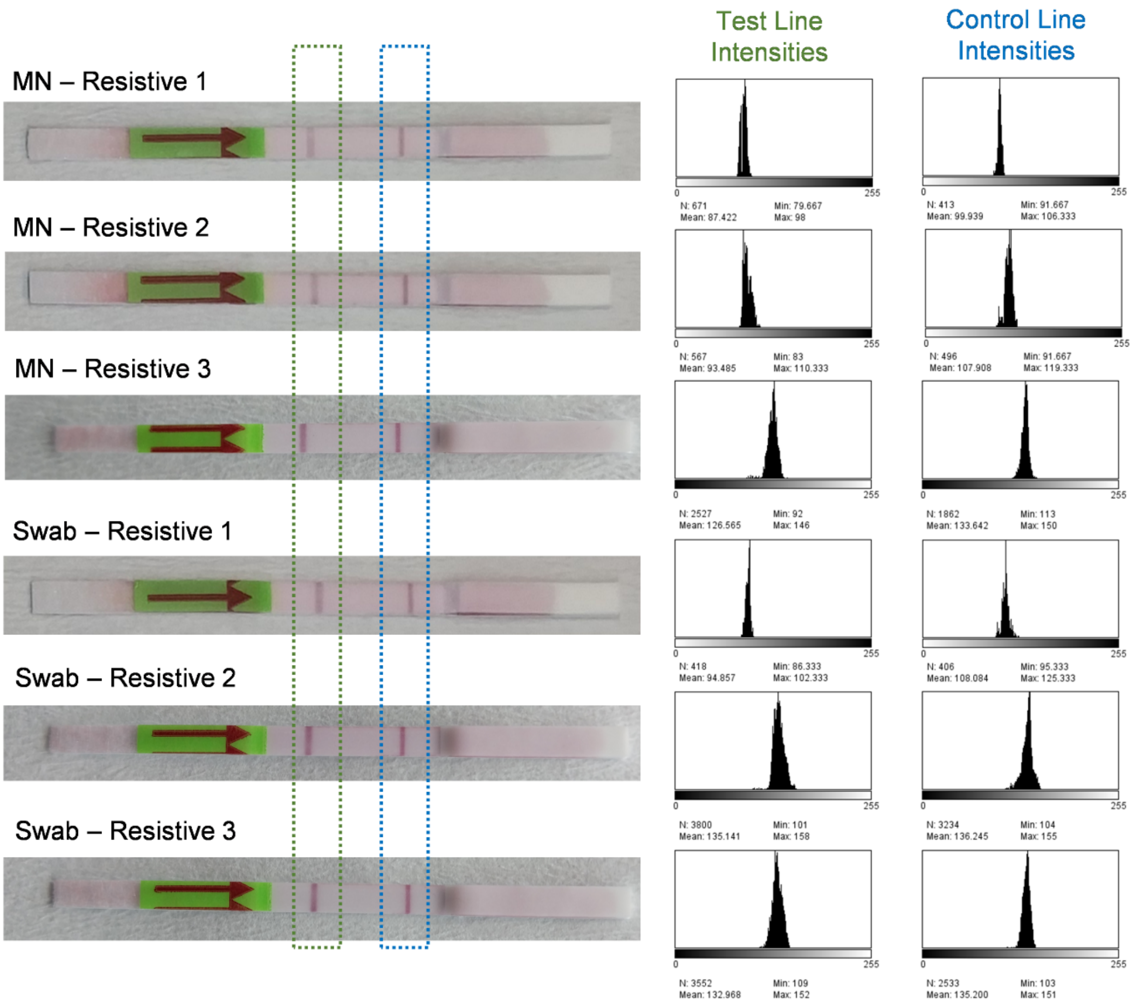

Figure S23. On the fifth day following inoculation, LFA test results for swab and MN samples from resistant leaves, including their intensity values, were presented.

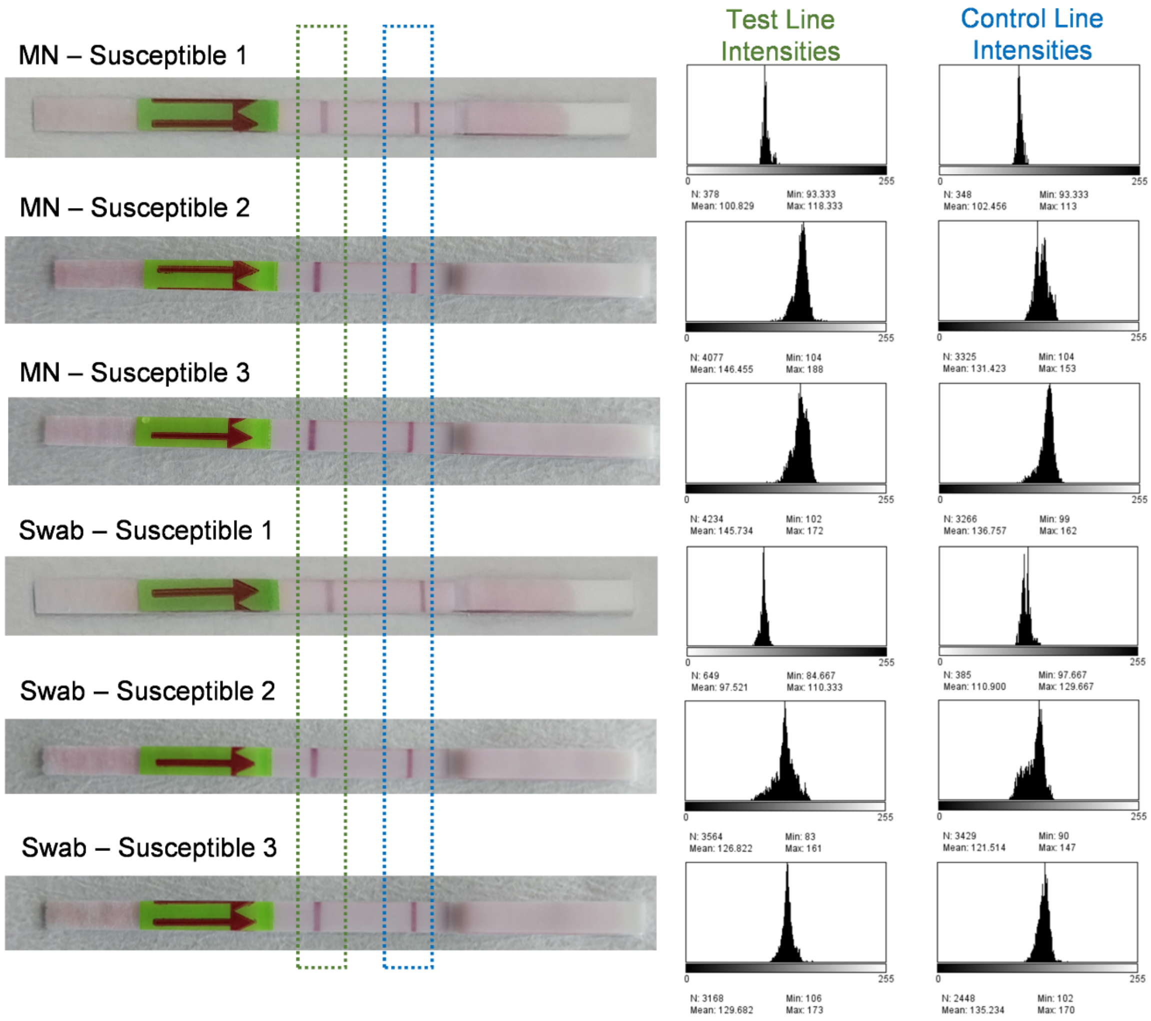

Figure S24. On day 15, LFA test results and intensity values for swab and MN samples from susceptible were evaluated using the bench incubator.

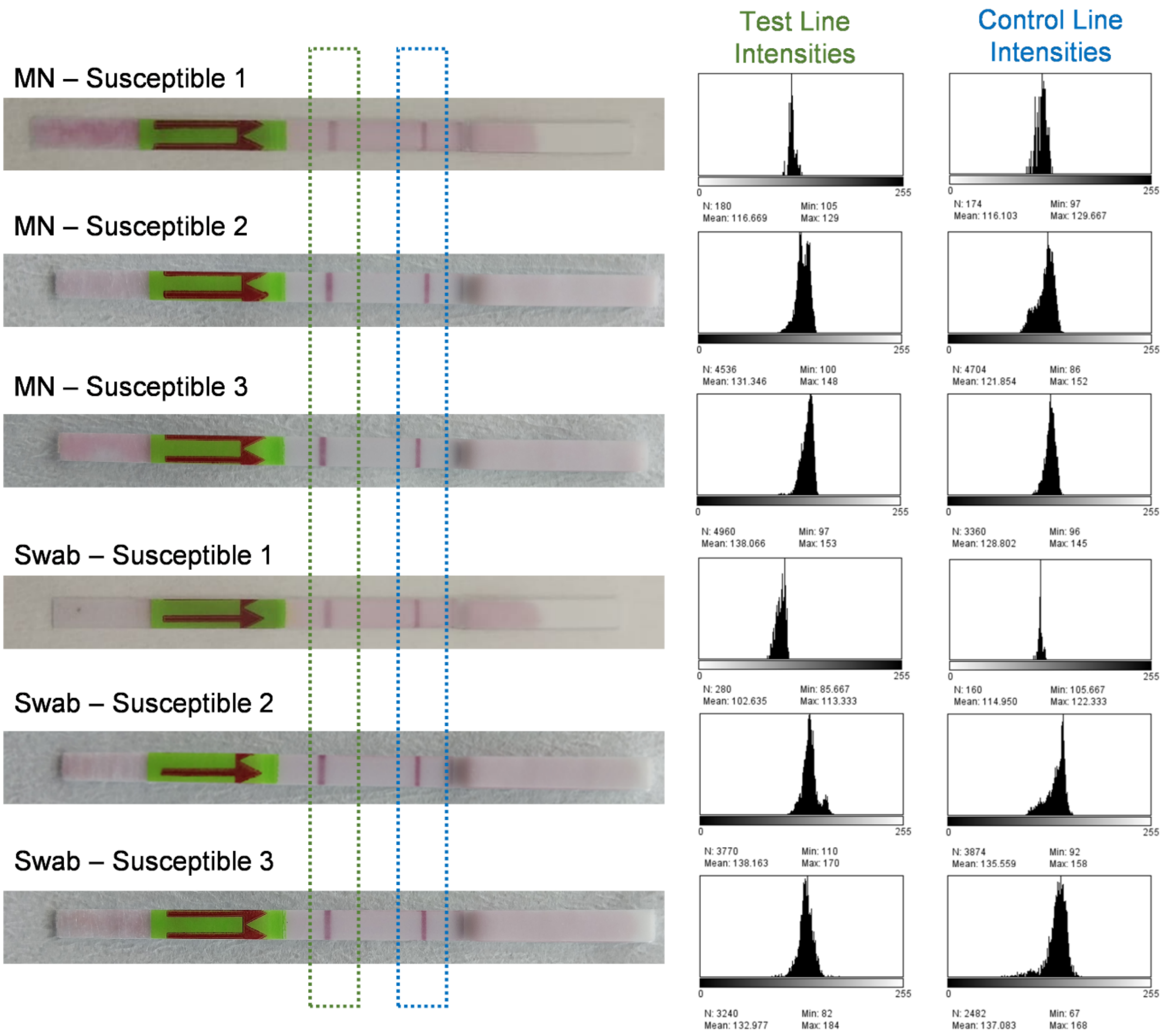

Figure S25. On day 15, LFA test results and intensity values for swab and MN samples from susceptible leaves were measured separately using our portable LAMP-LFA sensor system.

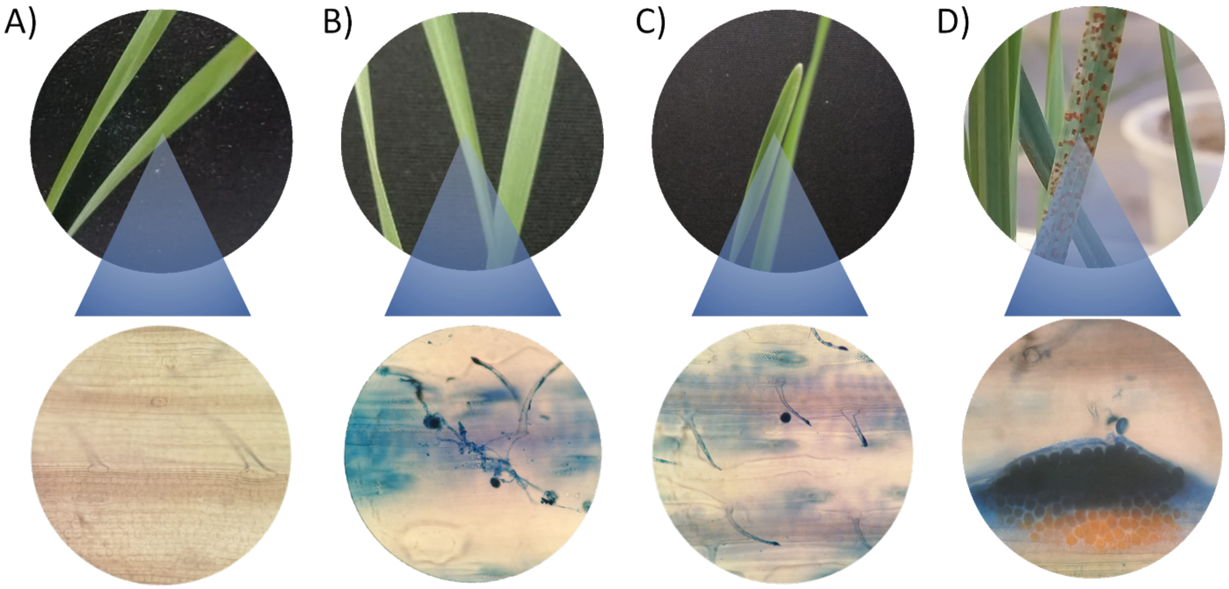

Figure S26. Microscope images were captured at 40× magnification to observe non-inoculated and inoculated susceptible and resistant leaves at 3 dpi. A) The non-inoculated leaves. B) Hyphael development of the spores was visible on the infected susceptible leaf. C) In contrast, the resistant leaf showed no hyphael development D) By the fifteenth day after inoculation, spore development was clearly evident on the susceptible leaf, indicating successful infection.

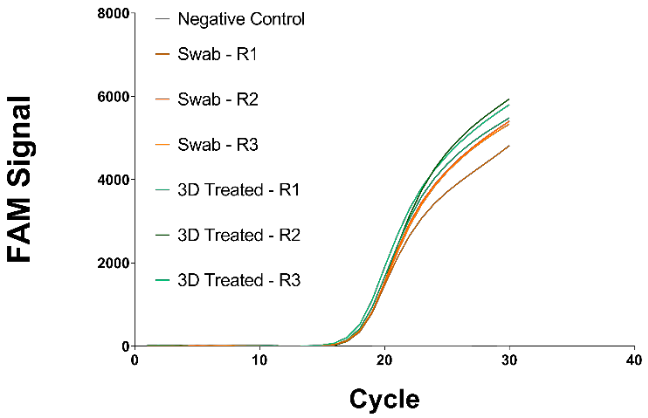

Figure S27. Real-time LAMP amplification curves comparing 3D-printed MN–exposed samples with non-exposed controls. Fluorescence intensity (FAM channel) is plotted against reaction time (minutes). The near-overlapping amplification profiles demonstrate that resin-fabricated MNs, following IPA cleaning and UV post-curing, did not introduce measurable inhibition to LAMP. Differences in Cq values remained within ~1% and endpoint fluorescence intensities within ~10%, consistent with normal replicate variation.

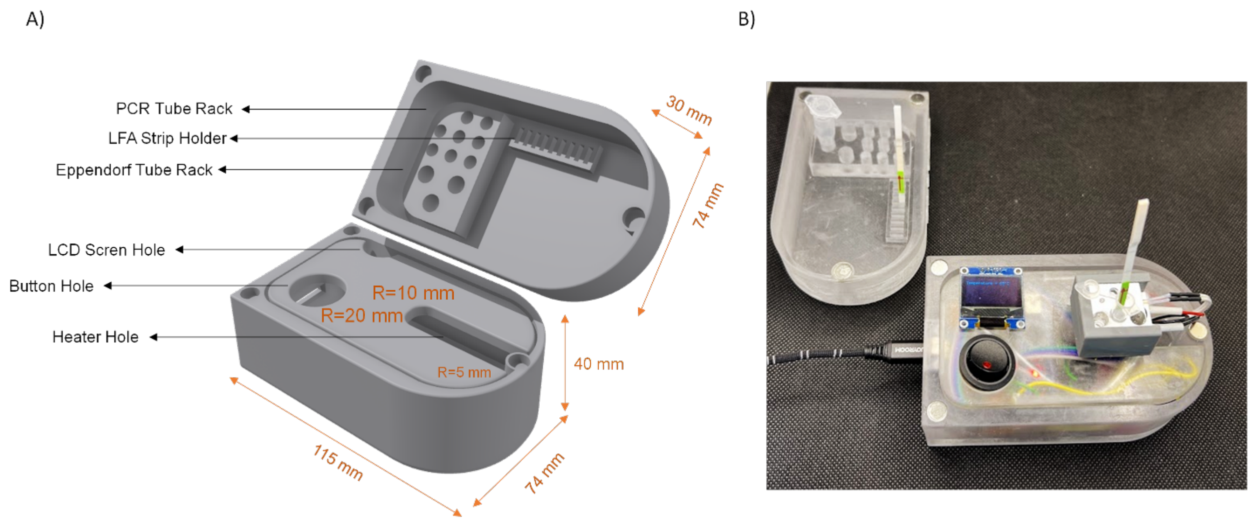

Figure S28. Design and geometrical features of the box used for portable LAMP system was demonstrated.

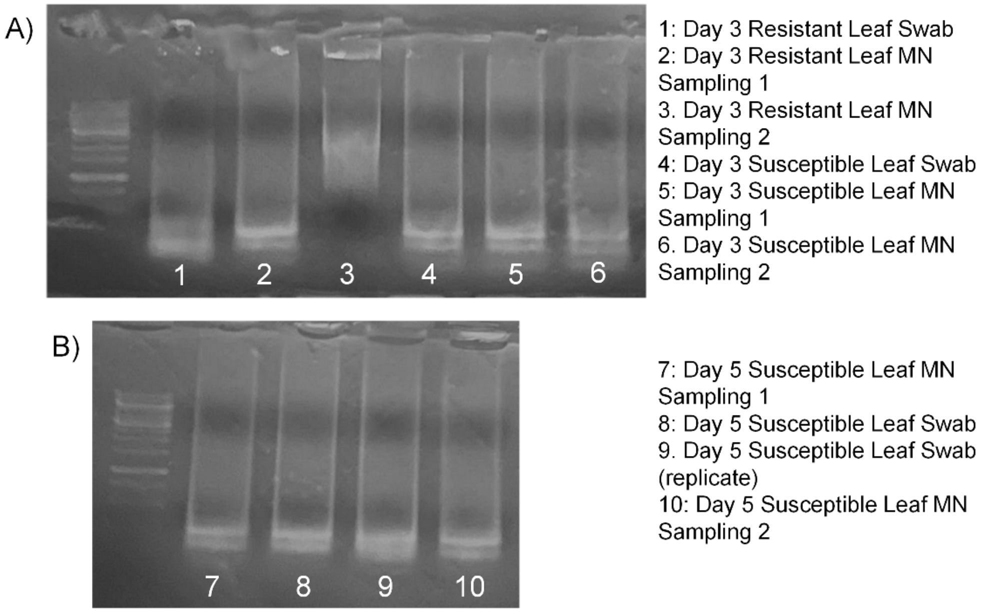

Figure S29. A) LAMP amplicons obtained from wheat leaves at 3 days post-inoculation, showing results from both susceptible and resistant leaves. Smear patterns characteristics of LAMP amplification are visible in the susceptible samples, while resistant leaves show lower amplification especially in MN sampling. B) LAMP amplicons from susceptible leaves at 5 days and healthy (inoculated) control leaves. Amplification smears are clearly observed in susceptible samples, whereas no amplification is detected in the healthy control.
